# Eco-friendly Cu–TiO_2_ nanoparticles from *Citrus limon* peel: integrated biological and computational evaluation

**DOI:** 10.1186/s13568-026-02060-2

**Published:** 2026-05-02

**Authors:** Hansa Gul, Zahida Nasreen, Muhammad Nauman Khan, Maged Mostafa Mahmoud, Mohammed S. Almuhayawi, Shadi A. Zakai, Tewekel Melese Gemechu, Muhammad Adnan, Alevcan Kaplan

**Affiliations:** 1 Department of Zoology, University of Mianwali, Mianwali, Punjab Pakistan; 2 Department of Botany, University of Chakwal, Chakwal, 48800 Punjab Pakistan; 3 EcoHealth Unit, King Fahd Medical Research Center, King Abdulaziz University, 21589 Jeddah, Saudi Arabia; 4 Department of Medical Laboratory Sciences, Faculty of Applied Medical Sciences, King Abdulaziz University, 21589 Jeddah, Saudi Arabia; 5 Department of Clinical Microbiology and Immunology, Faculty of Medicine, King Abdulaziz University, 21589 Jeddah, Saudi Arabia; 6 Department of Natural Resources Management, Ambo University, P.O. Box 19, Ambo, Ethiopia; 7 Department of Chemistry, Bacha Khan University, Charsadda, Khyber Pakhtunkhwa Pakistan; 8 Department of Pharmaceutical Botany, Faculty of Pharmacy, Dicle University, 21200 Diyarbakır, Turkey

**Keywords:** Cu–TiO_2_NPs, *Citrus limon* peel extract, Antibacterial activity, Anticancer activity, DFT, Molecular docking

## Abstract

The green synthesis of multifunctional nanomaterials from agricultural waste is a major target in sustainable biomedicine. This study reports the eco-friendly, one-pot synthesis of copper-doped titanium dioxide nanoparticles (Cu–TiO_2_NPs) using an aqueous peel extract of *Citrus limon* (Linn.) Burm. f. The formation of the nanoparticles was observed using the ultraviolet-visible spectroscopy, with an absorption edge below 400 nm with a redshift in the visible range (400–650 nm). X-ray diffraction (XRD) confirmed a mixed anatase-rutile crystalline phase, with an average crystallite size of 45.6 nm, and scanning electron microscopy (SEM) showed irregularly shaped agglomerated nanoparticles with an average diameter of 48 ± 0.46 nm. Field Emission Scanning Electron Microscopy (FESEM) revealed irregular, highly agglomerated nanoparticles with a primary size range of 49 ± 07 nm, while X-ray Photoelectron Spectroscopy (XPS) confirmed the oxidation states of Ti, O, and Cu, validating successful doping. Energy-Dispersive X-ray (EDX) analysis further confirmed uniform Cu incorporation into the TiO₂ lattice. In vitro testing showed a dose-dependent antibacterial effect against *Staphylococcus aureus* (ATCC 9144), *Staphylococcus epidermidis* (ATCC 12228), and *Pseudomonas aeruginosa* (ATCC 10145), with minimum inhibitory concentration (MIC) values ranging from 15 to 20 µg/mL. The highest zone of inhibition for Cu-TiO₂NPs was 21.00 ± 0.29 mm against *S. epidermidis* at 30 µg/mL, similar to the effect observed with ceftriaxone (30 µg/mL). The nanoparticles exhibited high antioxidant capacity, with a ferric reducing antioxidant potential of 67.5 ascorbic acid equivalents (AAE) µg/mL and a 54% 2,2-diphenyl-1-picrylhydrazyl (DPPH) scavenging capacity at 500 µg/mL. Cytotoxicity was measured using the 3-(4,5-dimethylthiazol-2-yl)-2,5-diphenyltetrazolium bromide assay, which indicated dose-dependent cytotoxicity in human hepatocellular carcinoma (HepG2) cells, with an IC_50_ of 150 µg/mL after 24 h. The stabilization of the L-ascorbic acid-Cu–TiO₂NPs complex was investigated using Density Functional Theory (DFT), and molecular docking revealed strong interactions with the target bacteria. This study uniquely integrates sustainable waste valorization, comprehensive in vitro bio evaluation, and computational validation of lemon peel-derived Cu-TiO_2_NPs for biomedical applications.

**Supplementary Information:**

The online version contains supplementary material available at 10.1186/s13568-026-02060-2.

## Introduction

The green synthesis of metal and metal oxide nanoparticles (MO-NPs) is increasingly considered more acceptable than conventional chemical methods because it is non-toxic, environmentally friendly, and sustainable (Chavan et al. 2026; Kedi et al. 2018; Muthuvel et al. 2020a, b). This method uses the different parts of plants, such as leaves, fruits, and peels, as reservoirs of secondary metabolites such as polyphenols and flavonoids which act as natural reducing and stabilizing agents (Dutta et al. 2022; Mohamed 2025; Mohamed et al. 2020). The recent developments in phytochemical-mediated nanotechnology indicate that adding copper to bimetallic and composite systems greatly increases their antioxidant, antimicrobial, and catalytic activities (Khan et al. 2025). Among these advanced materials, copper-doped titanium dioxide (Cu–TiO_2_NPs) is considered highly stable and safe (de Almeida et al. 2025). Doping TiO_2_ with copper (Cu) is a long-term strategy to improve its electronic characteristics and increase its reactive potential. Copper ions are inherently biocidal and enhance the antimicrobial ability of nanocomposites, making copper-doped TiO_2_ (Cu-TiO_2_) an excellent candidate for the next generation of materials (Mathew et al. 2018). Although the synthesis of Cu–TiO_2_NPs by conventional chemists is common, it usually requires hazardous reagents, significant energy, and generates toxic waste, leading to safety concerns (Sargazi et al. 2022). Although recent chemical methods have reduced these extreme temperature and energy requirements (Gibaud et al. 2025; Ramesh et al. 2015), green synthesis remains a more sustainable and safer method (Singh et al. 2018; Wang, 2004) (Firdhouse and Lalitha 2015). Recent mechanistic studies have further validated this green approach by demonstrating that primary plant metabolites, specifically sugars, can actively drive the synthesis of metal oxide nanoparticles at remarkably low temperatures (Elemike et al. 2019; Mohamed et al. 2025; Muthuvel et al. 2020a, b; Ramesh et al. 2015).

To achieve a truly circular economy, this study focuses on the valorization of agricultural waste using aqueous *Citrus limon* (lemon) peel extract. These peels contribute to about 44% of the wastes generated by processing juice extraction and are highly bioactive (containing D-limonene which is a strong natural surfactant) that aid in the controlled production of nanoparticles (Devarahosahalli et al. 2014; Maham et al. 2024).

There is an urgent need to develop novel, safe, and low-cost antimicrobial techniques because current traditional approaches often have high costs and limited efficiency (Singh et al. 2022). Nanoparticle-based systems offer a highly promising alternative, providing significant therapeutic potential through multiple mechanisms that are difficult for pathogens to resist (Dutta et al. 2024a, b; Sahu et al. 2023).

This study is innovative for its rare integration of sustainable synthesis, extensive biological evaluation, and computational validation. We present a one-pot green protocol for Cu-TiO_2_NPs, combining materials that are rarely associated with *C. limon* waste. In line with current research trends in research, this study employs an interdisciplinary approach that integrates molecular docking and biochemical dynamics to bridge the gap between traditional herbal knowledge and modern nanotechnology **(**Afzal et al. 2025). This Wet-lab and Dry-lab approach not only confirms the experimental data, but also gives an atomistic insight into how surface-bound phytochemicals can identify microbial and cancer-associated protein targets.

The novelty of this research is the specific utilization of *Citrus limon* Linn. Burm. f. peel for the green synthesis of Cu-TiO_2_NPs, coupled with comprehensive bioevaluation and integrated computational validation. Although green synthesis of TiO_2_NPs is established, the use of lemon peel extract for Cu-TiO_2_NPs and the detailed experimental and in silico correlation presented here have been less explored. While this approach offers sustainability, cost-effectiveness, and multifunctional properties, further in vivo studies and detailed phytochemical characterization are recommended to confirm efficacy, safety, and to identify the specific bioactive compounds responsible for nanoparticle formation and biological activity.

## Methods and materials

This was experimented at the Zoology Laboratory (MOSAEL) at the Department of Zoology, University of Mianwali, Punjab, Pakistan. *Citrus limon* peels were taken in a nearby village, the village of Sultan Wala Mochh, Tehsil and District Mianwali, Punjab Province, Pakistan. All chemicals and reagents that were used in this study were of analytical grade and were not purified further. Titanium isopropoxide [Ti{OCH(CH_3_)}_4_] (≥ 99% purity) was obtained from Sigma-Aldrich (Germany) and employed as the titanium source for nanoparticle synthesis. Copper(II) sulfate pentahydrate (CuSO_4_·5 H_2_O, ≥ 99% purity) was purchased from Merck (Germany) and used as the copper precursor for doping TiO_2_NPs. For antioxidant assays, 2,2-diphenyl-1-picrylhydrazyl (DPPH, ≥ 97%) and ascorbic acid (≥ 99%) were procured from Sigma-Aldrich. Ferric chloride (FeCl_3_, 99%) and potassium ferricyanide (K_3_Fe(CN)_6_, 99%), were also supplied by Merck (Germany). A 0.2 M sodium phosphate buffer (pH 6.6) was freshly prepared using analytical-grade reagents. Dimethyl sulfoxide (DMSO), used as a solvent in the UV-Vis and MTT assays, was likewise obtained from Sigma-Aldrich, while Mueller–Hinton agar (Bio-Rad US) was obtained from the Department of Zoology, University of Mianwali. The chemicals that were utilized in the research were of analytical grade and needed no extra purification. All the experimental procedures were carried out using deionized water (DH_2_O).

### **Preparation of*Citrus limon*peel extract**

After collection, *Citrus limon* peel samples were rinsed with deionized water (DH_2_O) to remove surface dust or impurities. The cleaned peels were cut into pieces and shade-dried at room temperature for 15 days. A 5 g portion of peel powder was mixed with 50 mL of distilled water (1:10 w/v) in a round-bottom flask, and the mixture was heated at 70 °C with constant stirring (150 rpm) for 1 h, as described by (Ali et al. 2020), with slight modifications. The extract was then stored at 4 °C for future use in NPs formation.

### **Cu-TiO**_**2**_**NPs synthesis**

**Fig. 1:**
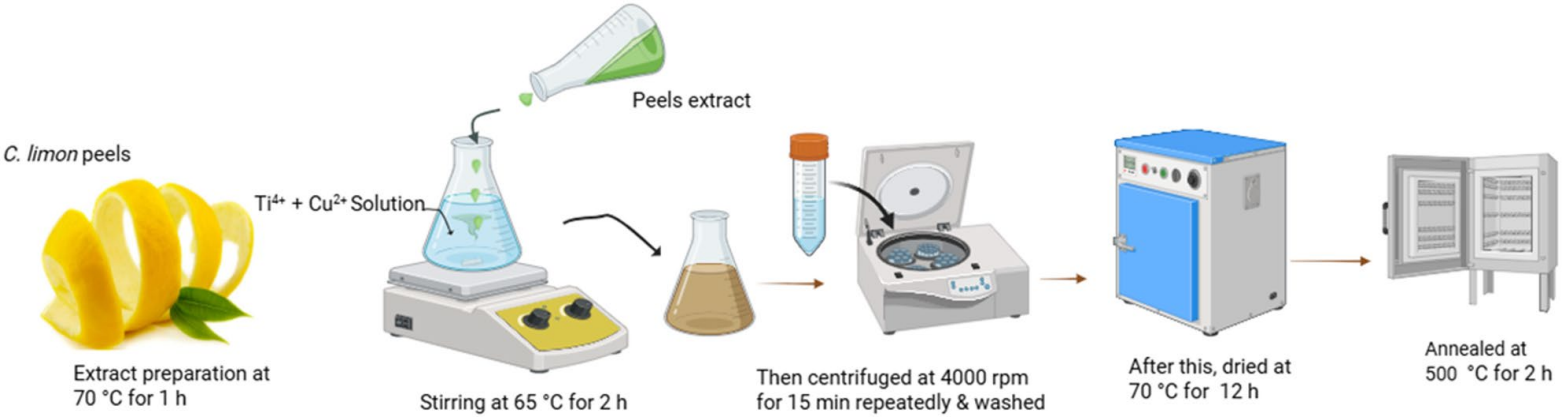
Schematic illustration of green synthesis of Cu-TiO2NPs

A Cu-TiO_2_NPs synthesis protocol (Ramzan et al. 2021) was followed with slight modifications. A 0.1 M titanium precursor solution was prepared by adding 3.03 mL of titanium isopropoxide [Ti{OCH(CH_3_)}_4_] dropwise into 100 mL of ice-cold distilled water (4 °C) under vigorous stirring. Uncontrolled hydrolysis of titanium isopropoxide was prevented by maintaining the reaction temperature at 4 °C and continuous stirring, which slowed the hydrolysis and promoted uniform nucleation (Mahshid et al. 2007). Separately, a 0.1 M copper solution was prepared by dissolving (2.497 g) of copper sulfate pentahydrate (CuSO_4_·5 H_2_O) in 100 mL of distilled water. A copper solution was then gradually placed in the already hydrolyzed titanium solution when stirred by stirring it to guarantee that the incorporation of the Cu^2+^ was homogeneous. *C. limon* peel extract (50 mL) was then added dropwise as a reducing and stabilizing agent to the titanium–copper mixture with constant stirring at 150 rpm for 2 h at 65 °C. The resulting suspension was centrifuged at 4000 rpm for 15 min, and the pellet was washed three times with deionized water. The material was dried at 70 °C for 12 h and then annealed at 500 °C for 2 h in a muffle furnace (Fig. 1). The annealing temperature of 500 C was selected to obtain crystalline anatase Cu-TiO_2_NPs and enable the incorporation of Cu^2+^ and avoiding the conversion of anatase to rutile as evidenced in the literature (BOZ 2024; Jarosz et al. 2015; Zhang and Wang 2016). The final Cu-TiO_2_NPs were collected and stored for further characterization.

### Characterization of Cu-TiO_2_NPs

Cu-TiO_2_NPs were thoroughly examined using different advanced methods. A Cecil Aquarius CE 7200 double-beam UV–visible spectrophotometer (UK) was used to obtain the UV–visible absorption spectra. Deionized water served as the dispersion medium for the nanoparticles to achieve a stable colloidal suspension for accurate measurements. Fourier-transform infrared (FTIR) spectroscopy was performed using a Shimadzu IRSpirit-T spectrometer with a diamond ATR accessory in the wavelength range of 400–4000 cm^− 1^ to identify the functional groups involved in nanoparticle formation and stabilization. The crystallinity and cleanliness of the synthesized Cu-TiO_2_NPs were determined using X-ray diffraction (XRD) with a JDX-3532 diffractometer from JEOL (Tokyo, Japan). Scanning electron microscopy (SEM) and energy-dispersive X-ray spectroscopy (EDX) were performed using a JEOL EDX system to analyze the surface features and composition. The surface morphology and particle size were investigated using field-emission scanning electron microscopy (FESEM, MIRA3 TESCAN) at an accelerating voltage of 12.0 kV. X-ray photoelectron spectroscopy (XPS) was conducted using a Thermo Scientific K-Alpha+ spectrometer, which employed a monochromatic Al Kα radiation source with energy of 1486.6 eV.

### Antibacterial assay

The antibacterial effects of Cu-TiO_2_NPs and the plant extract were evaluated against *Staphylococcus aureus* (ATCC 9144), *Staphylococcus epidermidis* (ATCC 12228), and *Pseudomonas aeruginosa* (ATCC 10145) by well diffusion, following the procedure of (Assad et al. 2024).

To perform the assay, 5.7 g of Mueller–Hinton (MH) agar was solubilized in 125 mL of distilled water. It was then autoclaved at 120 °C for 20 min under 15 psi to sterilize. The sterile medium (25 mL) was poured into Petri dishes and left to dry at room temperature for 20 min. Sterile cotton swabs and the streak plate method were used to inoculate each plate with the test bacterial strains. Four wells (6 mm in diameter) were punched into the inoculated agar surface using a sterile cork borer. A specific volume (50 µL) of the test solutions were added into the respective wells on each plate and labeled as follows: (a), -Ceftriaxone sodium (30 µg/mL; positive control), (b), Cu-TiO_2_NPs (30 µg/mL), (c), plant extract (30 µg/mL), and (d), distilled water as a negative control. The plates were incubated at 37 °C for 24 h, and the diameter of the zone of inhibition was calculated in millimeters. Triplicated experiments were done in a laminar flow cabinet using all the reagents and experimental procedures under aseptic conditions.

### Minimum inhibitory concentration (MIC) and Minimum bactericidal concentration (MBC) determination

The MIC and MBC of Cu-TiO_2_NPs against Gram-positive bacterial strains (*S. aureus*,* S. epidermidis*, and *P. aeruginosa*) were determined using the standard broth microdilution method. Briefly, serial dilutions of Cu-TiO_2_NPs in sterile Mueller-Hinton broth were prepared to achieve final concentrations of 5–40 µg/mL. A 100 µL aliquot of a freshly prepared bacterial suspension, adjusted to the 0.5 McFarland standard (~ 1 × 10^8^ CFU/mL), was inoculated into each dilution.

The microtubes were kept under sterile conditions with an incubation period at 37 °C for 24 h Bacterial growth was determined by use of turbidity after incubation. MIC was observed to be the minimum concentration of Cu-TiO_2_NPs that was not turbid.

To determine the MBC, 10 µL aliquots of tubes that had no any visible growth were subcultured on MuellerHinton agar and allowed to incubate at 37 °C for 24 h. The lowest concentration which gave no colonies on the agar plates was taken as the MBC. All experiments were repeated three times in order to make them reproducible.

### Anti-oxidant activity

#### DPPH free radical scavenging

The antioxidant potential of Cu–TiO_2_NPs synthesized using an extract was evaluated through the DPPH free radical scavenging assay following a modified protocol (Assad et al. 2024). Nanoparticles at various concentrations (100–500 µg/mL) were prepared and mixed with 100 µL of 0.1 mM DPPH solution. The mixtures were vortexed thoroughly and incubated in the dark at room temperature for 25 min. Ascorbic acid served as the standard reference antioxidant. After incubation, the absorbance of each sample was recorded at 517 nm using a UV–Vis Shimadzu UV-1800 spectrophotometer. The percentage of DPPH radical scavenging activity was calculated using Eq. (1).

1 $$\:\mathrm{\%}\:\mathrm{R}\mathrm{S}\mathrm{A}=\left(\frac{Abs\:of\:control\:517-Abs\:of\:sample\:517)\:}{Abs\:of\:control\:517}\right)\times\:100\:\:\:\:\:\:$$

Here, % RSA is the percentage Radical Scavenging Activity at Abs of control 517 and’ ‘Abs of sample 517’ refer to the absorbance values measured at 517 nm.

#### Ferric reducing antioxidant power (FRAP) assay

The ferric-reducing ability of the synthesized Cu–TiO_2_NPs was assessed using the FRAP method with slight modifications to the established protocol (Shobha et al. 2019). Nanoparticles were prepared in varying concentrations ranging from 100 to 500 µg/mL using distilled water. In the assay, a newly prepared FRAP reagent was employed, consisting of a blend of 300 mM acetate buffer at pH 3.6, a 10 mM solution of 2, 4, 6-tripyridyl-s-triazine (TPTZ) dissolved in 40 mM HCl, and 20 mM FeCl_3_·6 H_2_O, mixed in a 10:1:1 ratio. A volume of 100 µL of each nanoparticle sample was mixed with 3 mL of FRAP reagent and incubated at 37 °C in the dark for 30 min. The reduction of the ferric–TPTZ complex to the ferrous form produced an intense blue color, and the absorbance of each mixture was measured at 593 nm using a UV–Vis spectrophotometer. Ascorbic acid was used as the standard antioxidant for comparison. The FRAP assay quantifies reducing power based on a standard calibration curve. Therefore, the reducing power of the samples was expressed as ascorbic acid equivalents (µg/mL) derived from this calibration curve rather than a direct calculation equation.

#### Anticancer assay

Human hepatocellular carcinoma (HepG2) cells used in this study were obtained from the American Type Culture Collection (ATCC, VA, USA) and cultured in Dulbecco’s Modified Eagle Medium (DMEM) supplemented with 10% fetal bovine serum (FBS) and 1% penicillin- streptomycin solution. The cultures were incubated in a humidified incubator with 5% CO_2_ at 37 °C. After the cells reached 80–90% confluence, they were subcultured by detachment with trypsin-EDTA. Cytotoxicity assays were performed on cells at passages 3–10.

In the MTT cytotoxicity assay, 1 × 10^6^ HePG2 cells/mL were seeded into 96-well plates and allowed to adhere overnight. Different concentrations of Cu-TiO_2_NPs (10, 50, 100, 150, and 200 µg/mL) in Dulbecco’s Modified Eagle Medium (DMEM) were added to the cells. Untreated cells served as the negative control. After incubation for 24 h at 37 °C and 5% CO_2_, 20 µL 10 M of MTT solution (5 mg/mL) was added to each well. The plates were incubated for an additional 4 h to allow viable cells to metabolize MTT, resulting in the formation of purple formazan crystals. After this incubation, 100 µL of dimethyl sulfoxide (DMSO) was introduced into each well for dissolution of the formazan crystals. The solution was analyzed using a microplate ELISA reader, measuring at a wavelength of 540 nm, with a reference wavelength of 690 nm to eliminate background absorbance.

2 $${\text{Cell Viability (\%) = A1 /A0}} \times {\mathrm{100}} $$

A_1_ and A_0_ denote the absorbance measurements for the wells that have been treated and those that serve as untreated controls, respectively. The assays were performed in triplicate to ensure reproducibility.

### Statistical analysis

The data are presented as the average ± standard deviation. Statistical analyses and visualizations were conducted using Origin Pro version 2021 and Microsoft Excel 2016. For the antibacterial and antioxidant activity data, one-way analysis of variance followed by Tukey’s honest significant difference (HSD) post hoc test was used to determine significant differences between groups (*p* < 0.05).

## Computational study

### Nanoparticle model construction and surface engineering

The model was fabricated using a bottom-up structural engineering technique based on the construction of Cu-TiO_2_NPs. A geometrical template was derived from the CuTiO_2_ lattice framework, originally obtained as the bulk crystal structure of CuTiO (mp-1187469- Materials Project database) (Jain et al. 2020), which served as the geometrical template. Nanoclusters were created by generating a supercell and truncating it to form a finite nanocluster.

Substitutional doping of copper was performed by replacing a Ti atom with a Cu atom, which acted as the Cu dopant. The resulting Ti_8_CuO_x_ structure represents a model Cu-TiO_2_NPs. To simulate realistic surface chemistry under ambient aqueous conditions, undercoordinated surface oxygen atoms were protonated to form hydroxyl (OH) groups. This hydroxylation reaction removes hanging bonds, stabilizes the cluster electronically, and resembles the experimentally observed passivation of metal oxide nanoparticles at the surface. The substrate used in the adsorption and capping studies of L-ascorbic acid was the final hydroxylated Cu-TiO_2_NPs cluster (38 atoms) (Fig. 1S).

### Structural pre-optimization and conformational search

The initial geometries of pure L-ascorbic acid, Cu-TiO_2_NPs, and the resulting complex (L-ascorbic acid-Cu-TiO_2_NPs) were subjected to a rigorous two-stage pre-optimization protocol using the extended tight-binding (xTB) method. First, coarse structural relaxation was performed using with GFN-FF force field to resolve steric constraints (Helal 2025). Subsequently, a refined geometry optimization was conducted at the GFN2-xTB semi-empirical level (Bannwarth et al. 2019). To ensure a stable electronic ground state for the transition metal cluster, an unrestricted Hartree–Fock (UHF) approach with a spin multiplicity of 2 (doublet state) was utilized (Reusch and Grabert 2003). Electronic temperature effects were included via Fermi smearing at 5000 K. This elevated electronic temperature was applied solely to facilitate SCF convergence and did not represent a physical thermodynamic temperature. A relaxed accuracy threshold (α = 0.5) and 1000 SCC iterations were employed to facilitate convergence of the electronic density.

### Density functional theory (DFT) investigations

High-level electronic structure calculations were performed using the PySCF and gpu4pyscf software packages (Wu et al. 2025). The final geometry optimizations and electronic property evaluations were conducted using the PBE (Perdew-Burke-Ernzerhof) generalized gradient approximation (GGA) functional (Perdew et al. 1996). The def2-SVP basis set was employed for all atomic species to ensure a balanced description of the localized electronic environments of the Ti and Cu atoms (Hellweg and Rappoport 2015). To accurately quantify the long-range non-covalent interactions between ascorbic acid and the nanoparticle surface, Grimme’s dispersion correction (Becke-Johnson damping (D3BJ) was implemented (Schröder et al. 2015).

Given the 465-electron count of the system, an unrestricted Kohn-Sham (UKS) formalism was adopted in the doublet state (spin multiplicity 2 S + 1 = 2) (Gritsenko and Baerends 2004). To handle the high density of states inherent to the CuTiO cluster, Fermi smearing with a sigma value of 0.01 Hartree was utilized. All structures were optimized until the energy and gradient convergence thresholds were satisfied within 10^− 6^ Hartree and 10^− 3^ Hartree/Bohr, respectively.

### Binding energy and electronic property analysis

The thermodynamic stability of the nanoconjugate was quantified by calculating the binding energy (*Ebinding*​) according to the following equation:

3 $$ E_{{binding}} = E_{{complex}} - {\text{ }}(E_{{NP}} + E_{{ligand}} ) $$

where *E*_*complex*,_ *E*_*NP*_ and *E*_*ligand*_​ correspond to the total electronic energies of the capped nanoparticle, isolated Cu-TiO_2_NPs, and L-ascorbic acid, respectively. Additionally, the electronic coupling and chemical reactivity of the system were evaluated by examining the highest occupied molecular orbital (HOMO), the lowest unoccupied molecular orbital (LUMO), and the energy gap (*Eg*​) between HOMO and LUMO. Volumetric data for these frontier molecular orbitals were generated using cubegen integration to visually map the isosurfaces. Multiwfn (version 2026.2.2) was utilized to generate the projected density of states (PDOS) and reduced density gradient (RDG) plots (Dutta et al. 2024a, b).

### Molecular docking

**Fig. 2:**
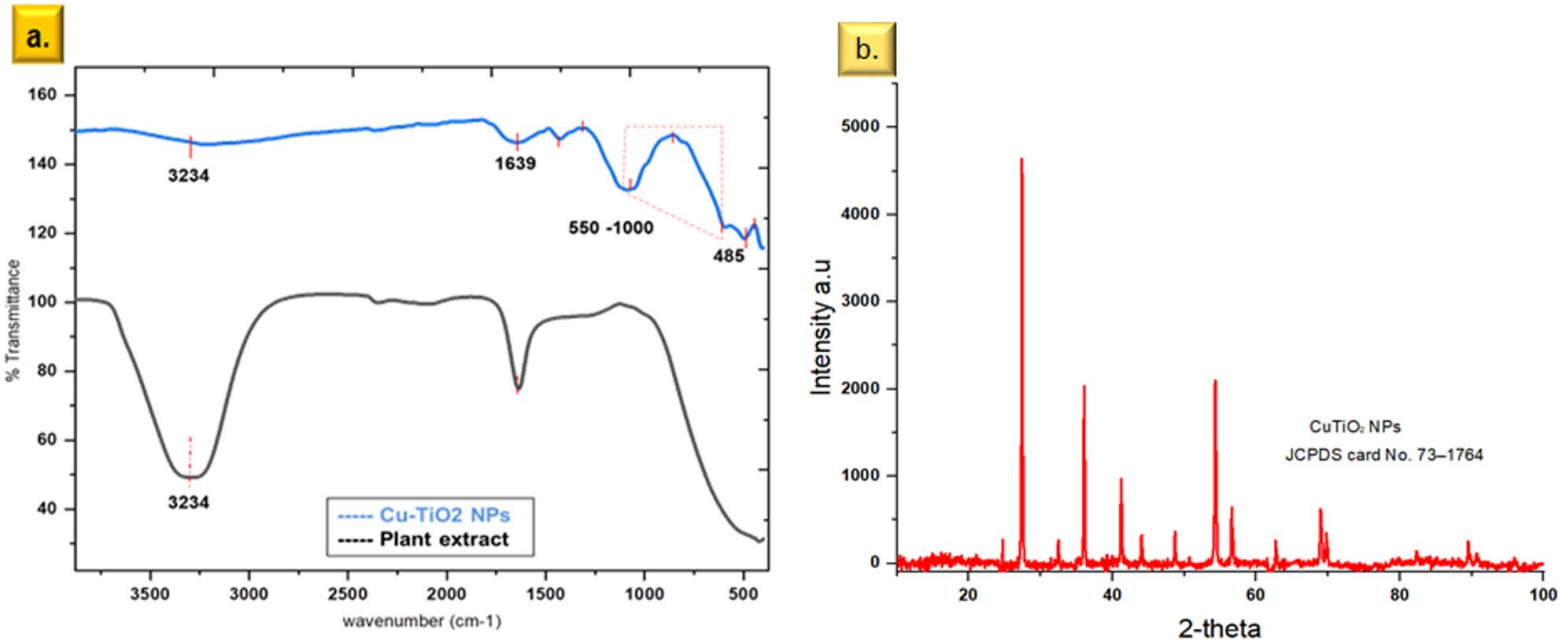
**a** FTIR pattern of biosynthesized Cu-TiO_2_NPs, **b** XRD pattern of biosynthesized Cu-TiO_2_NPs

To simulate realistic bio-interactions, docking was performed using the DFT-optimized L-ascorbic acid-Cu-TiO_2_NPs nanoconjugate as the ligand, representing the “phytochemical corona (Dutta et al. 2024a, b). To evaluate the molecular interactions of Cu-TiO₂NPs with biological systems, the crystal structures of specific protein targets were retrieved from the RCSB Protein Data Bank (https://www.rcsb.org/) (Garrido-Palazuelos et al. 2024a, b). The targets were selected based on their fundamental functions in microbial pathogenesis and human therapeutic pathways (Fig. 2S).

DNA gyrase B (GYRB) of *S. aureus* (PDB ID: 3TTZ) was selected as the antibacterial target because it plays a significant role in DNA topology during replication (Sherer et al. 2011). Polymerization of the cell division protein is critical for bacterial cytokinesis; therefore, this protein was chosen. Glucose-1-phosphate thymidylyltransferase (RmlA) (PDB ID: 4B4B) of *P.* aeruginosa was targeted because it is essential for the synthesis of l-rhamnose, a crucial.

component of the bacterial cell wall (Alphey et al. 2013). Moreover, the AmiE region of Autolysin E (atlE) of *S. epidermidis* (PDB ID: 3LAT) was targeted because it plays a central role in surface adhesion and biofilm formation (Vemula et al. 2023; Zoll et al. 2010). Human peroxiredoxin-5 (PRDX5, PDB ID: 1HD2) was chosen to evaluate its antioxidant potential as it can detoxify reactive peroxides (Declercq et al. 2001). Finally, caspase-3 (PDB ID: 1CP3) was identified as a major target to be explored to understand the apoptotic induction cascades underlying the observed anticancer effect (Srivastava and Saxena 2023). All unnecessary molecules, such as water, ions, and bound ligands, were removed to prepare the receptors for docking. Following this, the proteins were transformed into PDBQT format, with polar hydrogen atoms and Kollman charges incorporated using AutoDock Tools (Garrido-Palazuelos et al. 2024a, b; Gul et al. 2026; Rose et al. 2016).

Molecular simulations of docking were carried out to assess the interactions of the five biological targets proteins with the selected ligands. The Lennard-Jones parameters used in the Standard AutoDock 4.2 are not sufficient to describe the metal-ligand distances in active sites of metalloproteins. Thus, to achieve crystallographically determined coordination distances, custom parameters were created to Cu(II) and Ti(IV). The atoms coordinating the metals (N, O, and S to the metal centers (3.00 Å) were given the specific atom types with modified van der Waals radii. For Cu(II), parameters were calibrated to reproduce experimental Cu–N (2.00 Å), Cu–O (1.95 Å), and Cu–S (2.15 Å) coordination distances from protein crystal structure surveys (Harding 2001; Rulíšek and Vondrášek 1998). For Ti(IV), parameters were derived from the Universal Force Field (Rappé et al. 1992) and refined to match typical Ti–O (1.95 Å) and Ti–N (2.10 Å) coordination distances observed in titanium-containing complexes. All parameters were validated using Lorentz–Berthelot combining rules (Table 1S).

Molecular docking simulations were performed utilizing the AutoDock 4.2.6 engine (Morris et al. 2008). Affinity maps were generated with AutoGrid 4, using a grid box of 60 × 60 × 60 points and a spacing of 0.375 Å centered on the known active sites (Table 2S). Docking employed the Lamarckian genetic algorithm (LGA) (Fuhrmann et al. 2010). To preserve the structural integrity of the nanoparticle–ligand complex, as determined by DFT, the ligands were treated as rigid bodies. Ten independent docking runs were performed per ligand with a population size of 150 and 2,500,000 energy evaluations per run. Post-docking analysis was conducted by clustering the results with a 2.0 Å RMSD tolerance. The best-binding poses were selected based on the lowest free energy of binding (ΔG). Interaction analysis was performed using PLIP v2.4.0 to examine the residues within the binding pocket (Adasme et al. 2021).

## Results

### UV analysis

**Fig. 3:**
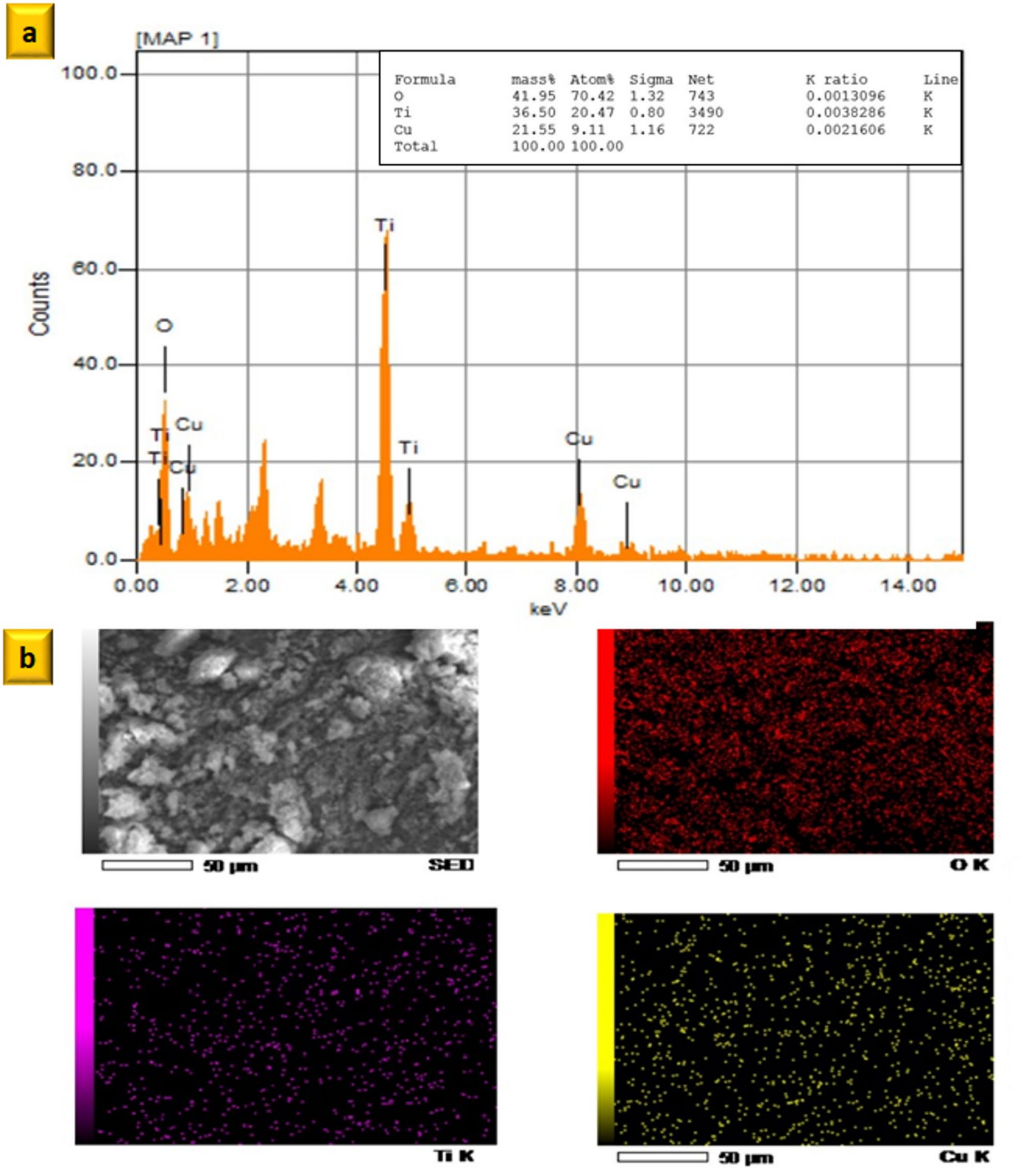
Elemental analysis of the biosynthesized Cu-TiO_2_NPs: **a** EDX spectrum showing the elemental composition with quantitative weight and atomic percentages. **b** Elemental mapping images that verify the even distribution of Titanium (Ti) (Purple), Oxygen (O) (Red), and Copper (Cu) (Yellow)

The UV-Vis spectrum of the synthesized Cu-TiO_2_NPs shows a sharp absorption edge below 400 nm, confirming the TiO_2_ semiconductor framework. A pronounced redshift and extended absorption into the visible region (400–650 nm) indicate successful incorporation of copper dopants (Fig. 3S).

### FTIR analysis

The FTIR spectra (Fig. 2a) were analyzed to identify the functional groups responsible for the reduction and stabilization of the nanoparticles. The *C. limon* extract (black line) shows a strong, broad absorption band at 3234 cm^− 1^ corresponding to O–H stretching of polyphenols, and a sharp absorption band at 1639 cm^− 1^ corresponding to C=O stretching of flavonoids. In the Cu-TiO_2_NPs spectrum (blue line), the intensity of the O–H band is much lesser and broader, and the carbonyl peak is shifted. These changes indicate that the phenolic hydroxyl and carbonyl functionalities of the extract are directly involved in binding to the surface of the Cu–TiO_2_NPs. According to the analysis developed by Dutta et al. (2019–2020), these alcoholic and phenolic groups are the main stabilizing agents (bio-corona), which allow nanoparticles to repel one another (Dutta et al. 2019, 2020a, b, c).

Notably, the peaks observed at approximately 485 and 580 − 540 cm^− 1^ correspond to Ti–O–Ti and Cu–O–Ti vibrational modes, respectively, confirming the successful incorporation of copper into the TiO_2_ lattice (Isa 2024). According to another study, the peak around 500–1000 cm^− 1^ is associated with Cu-TiO_2_NPs (Rajamannan et al. 2014) and the range 630–830 cm^− 1^ is also reported (Kukkola et al. 2011). A peak at 844 cm^− 1^ is attributed to Ti–O bending vibrations, while the absorption band at 1639 cm^− 1^ corresponds to TiO-OH (Dhamale et al. 2025) (Chougala et al. 2017). The broad band detected at approximately 3234 cm^− 1^ is indicative of O–H stretching vibrations, signifying the existence of surface hydroxyl groups (Ramírez-Estrada et al. 2022). Introducing copper into TiO_2_ results in the formation of localized states within the band gap, which enhances the absorption of visible light and reduces electron-hole recombination. This alteration can significantly boost the photocatalytic performance of the material, particularly in breaking down organic pollutants.

### XRD analysis

The crystal structure of Cu-TiO_2_NPs was analyzed using X-ray diffraction (XRD), as shown in Fig. 2b. The diffraction pattern revealed characteristic peaks corresponding to the (101), (004), (200), (105), (211), (204), (116), (220), and (215) planes, which are attributed to the anatase phase of TiO_2_ in accordance with Joint Committee on Powder Diffraction Standards (JCPDS) card No. 73–1764. Also, the fact that the material contains peaks which are indexed to the (101), (110), (111), and (220) planes point to the coexistence of the rutile phase (JCPDS card No. 781510) which proves the presence of the mixed-phase TiO2 structure. Notably, no distinct peaks related to CuO were observed, suggesting that copper ions were successfully incorporated into the TiO_2_ lattice rather than forming separate CuO phases. This is consistent with the literature (Teleki et al. 2008), where no Cu or CuO/Cu_2_O peaks appear at (5–10%) doping levels. Therefore, it is correct to show only TiO₂ peaks, and separate Cu reference peaks are not necessary. The broadening of diffraction peaks after Cu doping suggests changes in crystallite size and possible lattice expansion (Hesemans et al. 2023). Additionally, the peak at 25.34^0^ is for Cu-TiO_2_NPs anatase (Isa, 2024) as Garg et al., 2017 observed that it is also affiliated with the Miller index (101) (Garg et al. 2017; Sukhadeve et al. 2022). The average crystallite size of Cu-TiO_2_NPs, calculated using the Debye–Scherrer equation, was found to be 45.6 nm.

### EDX with elemental mapping analysis of Cu-TiO_2_NPs

Energy dispersive X-ray spectroscopy (EDX) and elemental mapping were employed to determine the elemental composition and confirm the successful doping of copper in the green-synthesized Cu-TiO_2_NPs (Fig. 3). The EDX spectrum confirmed the presence of titanium (Ti), oxygen (O), and copper (Cu) as the major elemental constituents of the nanoparticles. Quantitative EDX analysis revealed the sample contains 41.95 wt% O, 36.50 wt% Ti, and 21.55 wt% Cu, corresponding to atomic percentages of 70.42% O, 20.47% Ti, and 9.11% Cu. The significant incorporation of copper, both by weight and atomic percentages, suggests that Cu is not merely adsorbed on the surface or present as a secondary phase, but has been successfully (~ 22%) doped into the TiO_2_ matrix (Fig. 3a).

Additional information was obtained by mapping the elements which revealed that Ti, O, and Cu signals are strongly co-localized in the same spatial regions (Fig. 3b). This homogeneous distribution proves that copper is uniformly distributed within the framework of the TiO_2_ nanostructure, suggesting that copper is effectively substituted or interstitially doped into the titanium oxide lattice (Rao and Satyanarayana 2019; Ren et al. 2021; Sahu et al. 2018). Notably, discrete groups or separate areas of copper oxide were not observed, eliminating the possibility of simple physical mixing or surface decoration. The high atomic percentage of oxygen further supports the formation of stoichiometric or oxygen-rich metal oxide frameworks (Azizi et al. 2014).

Norris et al. (2008) reported that introducing copper as a dopant can alter the morphology, dimensions, and electronic properties of a photocatalyst. Energy dispersive X-ray diffraction (EDX) analysis showed considerable percentages of Ti, O, and Cu in the structure of the as-prepared Cu-TiO_2_ catalyst. In addition, the distribution of elements with colored dots in the mapping images confirms the presence of Cu in the Cu-TiO_2_ product.

### Scanning electron microscopy (SEM)

**Fig. 4:**
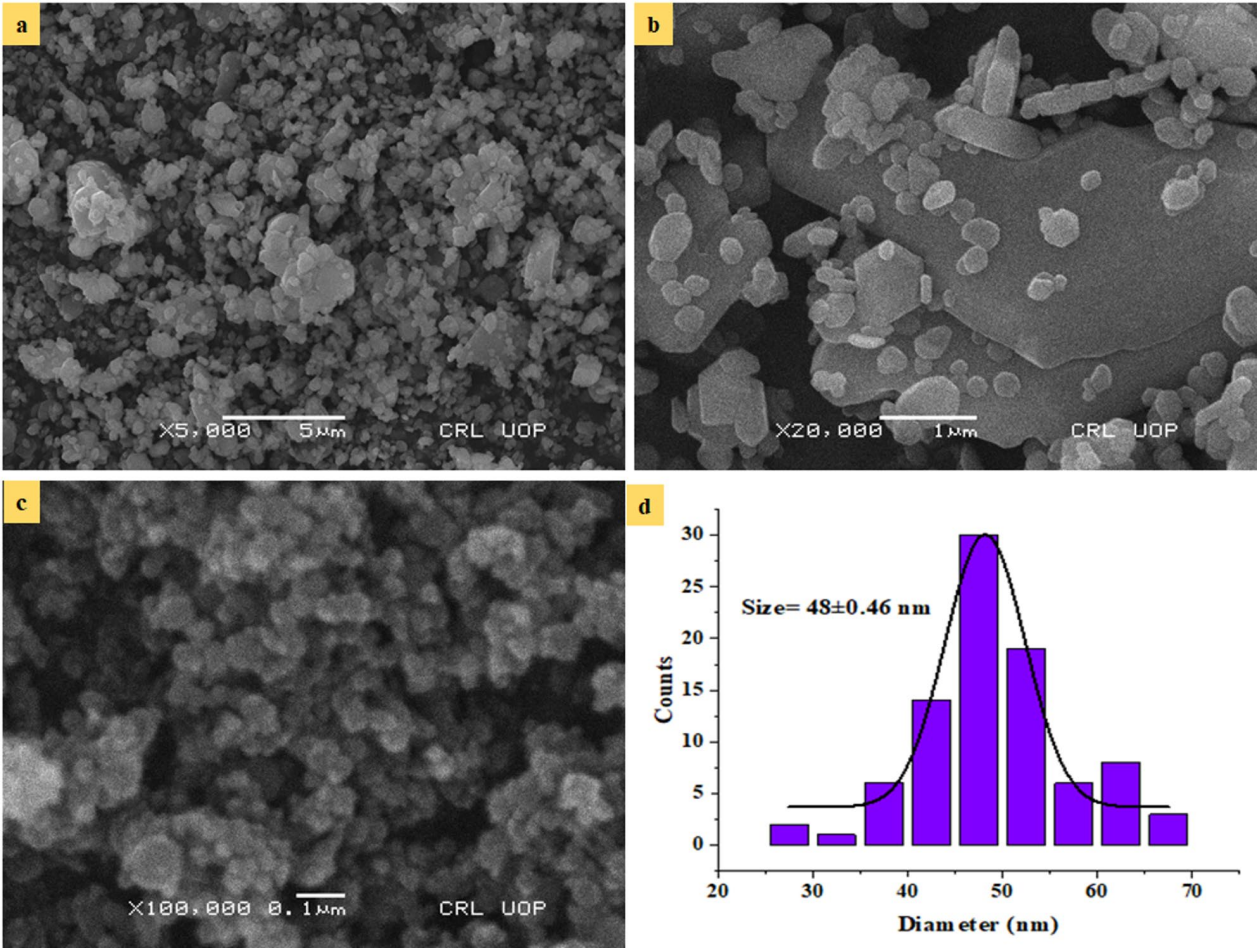
The morphology and microstructural features of the synthesized material were examined (SEM); Micrographs at **a** 5,000×, **b** 20,000×, and **c** 100,000× magnification show a heterogeneous morphology consisting of agglomerated, quasi-spherical nanoparticles (**d**) Distribution Histogram

The green-synthesized Cu-TiO_2_NPs were thoroughly analyzed for their surface morphology and elemental distribution using scanning electron microscopy (SEM) in conjunction with energy dispersive X-ray spectroscopy (EDX) (Jalali et al. 2023) (Fig. 4). The SEM micrographs, at various magnifications (Fig. 4a-c) provide give a detailed examination of the material’s structural features. At the lower magnification of 5,000x (Fig. 4a), the material appears to consist of numerous particles forming larger, irregular agglomerates. On the 20,000x magnification (Fig. 4b), a heterogeneous appearance is seen, with smarter, slightly spherical nanoparticles on one hand, and larger, plate-like microarchitecture, on the other. Figure 4c is the highest magnification (100, 000x) of the nanostructure which depicts a high concentration of quasi-spherical nanoparticles. Although the degree of aggregation is quite high, the single particles can be differentiated easily. The images of the SEM were used to create a size distribution histogram (Fig. 4d) to estimate the size of the particles. A fit to a Gaussian curve was done on the data, which gave a mean nanoparticle diameter of 48 ± 0.46 nm. The histogram displays rather a narrow distribution with the majority of the particles in the 40–60 nm range. This implies that the synthesis technique employed in this study is effective in the manufacture of nanoparticles in a narrow size range, which is similar to other ecofriendly synthesis techniques. The SEM micrograph showed that there was a cluster of agglomerated nanoparticles whose morphology was irregular and that they had rough and porous surfaces. It means that the surface area to volume ratio in this structure is high, which is beneficial in processes like catalysis and sensing (Hkiri et al. 2023).

### Field emission scanning electron microscopy (FESEM)

The surface morphology of the synthesized Cu-TiO_2_NPs was analyzed using FESEM (MIRA3 TESCAN). The micrographs in Fig. 4S(a-b) show that the nanoparticles predominantly exhibit quasi-spherical and irregular shapes. When the magnification is reduced (10,000×), Cu-TiO_2_NPs are presented as chunky secondary aggregates and massive aggregates. The surface topography of such aggregates shows that it has a very granular and textured surface which is typical of the doped metal oxide nanostructures, as reported in the literature (Ghotekar et al. 2024).

At higher magnification (70,000 x) the micrographs indicate that these large aggregates are made of closely packed primary nanoparticles. The sizes were measured through a generation of a size distribution histogram (Fig. 4S(c)). A graphic fitting was made to the data using a Gaussian curve and the results of the curve showed that the major nanoparticles have an average diameter of 49 + 07 nm. This value is highly similar to both the XRD and SEM values of crystallite size (45.6 nm and 48 nm, respectively). Despite a certain agglomeration of the Cu-TiO_2_NPs, probably because of interparticle van der Waals forces, distinct primary particles are easily identified within the clusters.

This is because of a high surface to volume ratio created by the presence of a textured surface and the creation of porous interstitial networks within the secondary clusters. The reason why this morphology is important is because it enables enhanced interactions with the bacterial cell walls or chemical reactants. All in all, the FESEM observations have confirmed the effective preparation of nanostructured Cu-TiO_2_NPs with a major particle size somewhere below 50 nm, which is applicable in advanced functional applications.

### X-ray photoelectron spectroscopy (XPS) spectra

**Fig. 5:**
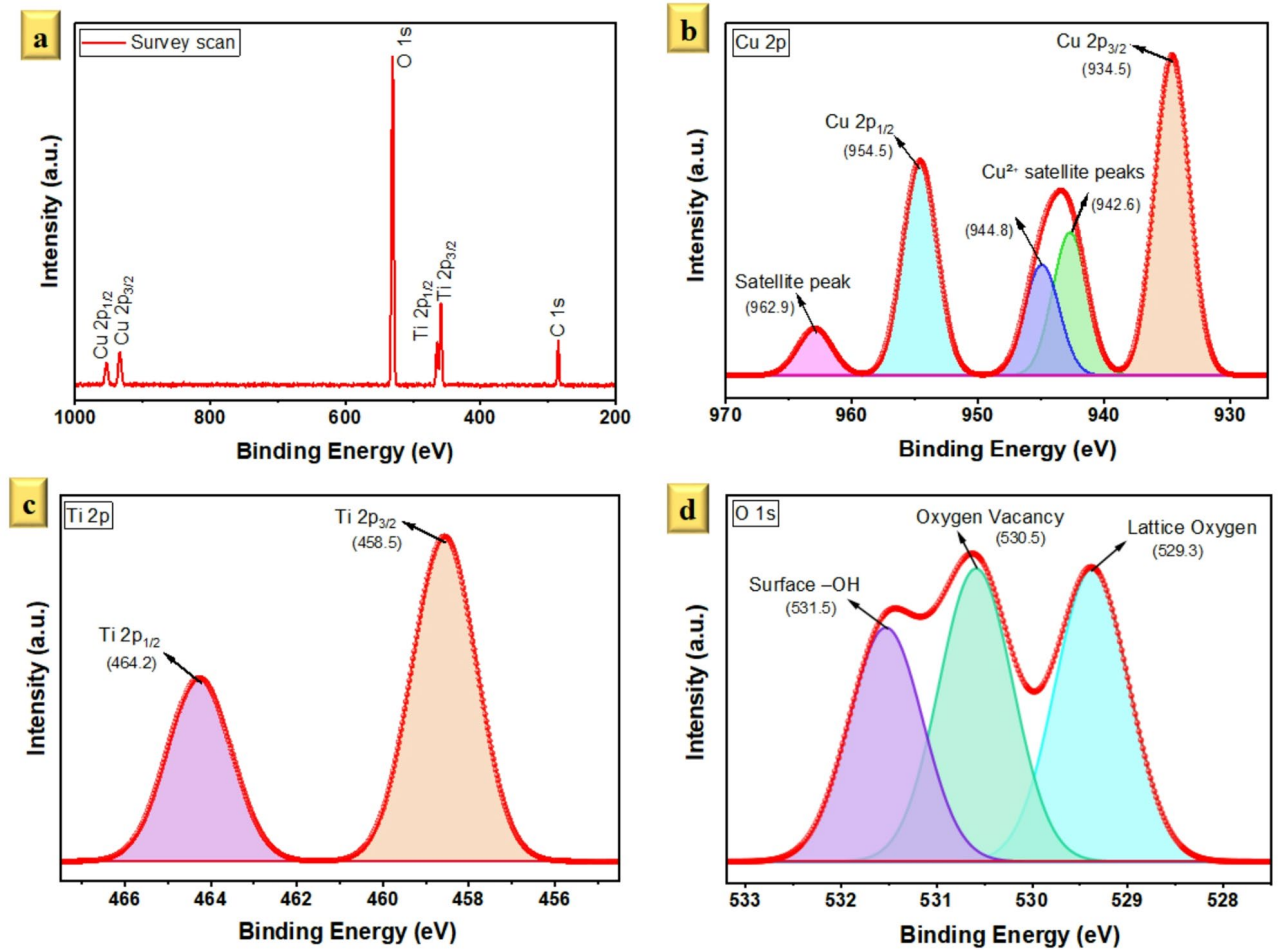
**a** Wide survey spectrum; **b** High-resolution Ti 2p spectrum confirming Ti^4+^; **c** O 1s spectrum deconvoluted into lattice oxygen, vacancies, and hydroxyl groups; **d** Cu 2p spectrum confirming the Cu^2+^ oxidation state via satellite peaks

XPS was employed to analyze the chemical composition and elemental oxidation state of the Cu- TiO_2_NPs. Figure 5(a) has a spectrum of the survey illustrating that there are Ti, O and Cu in the sample. The peak at C 1s (around 284.8 eV) is taken to be adventitious carbon contamination either of the instrument or the conditions and was the one to be used to calibrate the binding energy. The high-resolution Ti 2p spectrum is presented in Fig. 5(b). The $\:Ti\text{}2{p}_{3/2}$ and $\:Ti\text{}2{p}_{1/2}$ core levels were identified at 458.5 and 464.2 eV, respectively, and two characteristic peaks were observed. The spin-orbit separation of 5.7 eV between these two peaks is comparable to the values recorded for $\:T{i}^{4+}$ in a $\:Ti{O}_{2}$ lattice in the $\:T{i}^{4+}$ oxidation state (Cao et al. 2017). Figure 5(c) indicates that the O 1s spectrum can be deconvoluted into three separate peaks to determine the various oxygen species. The peak at 529.3 eV corresponds to lattice oxygen ($\:{O}^{2-}$), which is part of the $\:Ti$-$\:O$ crystal structure of $\:Ti{O}_{2}$. The peak at 530.5 eV is attributed to oxygen vacancies ($\:{O}_{vac}$) or oxygen atoms near defects in the lattice (Zhang et al. 2017). In addition, the surface hydroxyl groups (–OH) are attributed to the peak at 531.5 eV (Wang et al. 2014). The presence of a strong peak for oxygen vacancies indicates that doping with Cu is effective in producing structural defects, which is important for capturing photogenerated charges.

Another chemical investigation was conducted using the Cu 2p spectrum (Fig. 5d) to identify chemical states of the Cu dopant. The primary peaks at 934.5 and 954.5 eV are attributed to $\:Cu\text{}2{p}_{3/2}$ and $\:Cu\text{}2{p}_{1/2}$ respectively. Notably, there are strong shake-up satellite peaks at 942.6, 944.8, and 962.9 eV. This observation is consistent with the literature (Pedroza- (Tang et al. 2021), confirming that copper exists in the $\:C{u}^{2+}$ oxidation state. These strong satellite features are an absolute fingerprint of the $\:C{u}^{2+}$ oxidation state (Bashiri et al. 2017). The findings show that copper largely exists in the + 2 oxidation state, probably replacing $\:T{i}^{4+}$ in the lattice, or that copper oxides on the $\:Ti{O}_{2}$ surface are highly dispersed (Córdoba et al. 1998).

### Antibacterial activity

**Fig. 6:**
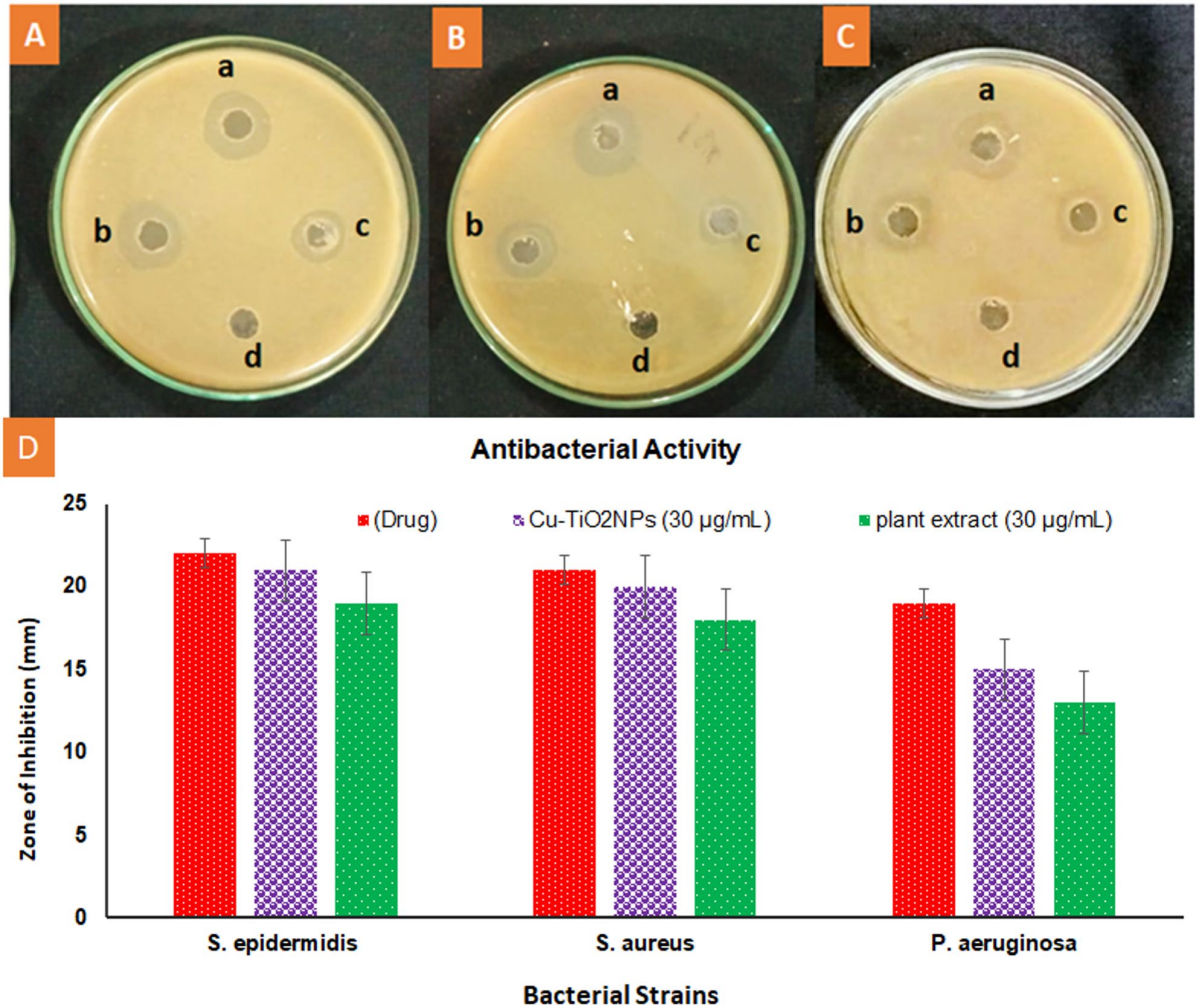
Antibacterial efficacy of Cu-TiO_2_NPs against, *S. epidermidis*,* S. aureus* and *P. aeruginosa* bacterial strains (**A**-**C**) (a) Ceftriaxone sodium (30 µg/mL; positive control), (b) Cu-TiO_2_NPs (30 µg/mL) (c) Plant extract (30 µg/mL), and (d) Deionized water (30 µg/mL) show no zone of inhabitation, (**D**) Graphical representation

Traditional antibiotics function by targeting specific molecular positions on or within the bacterial membrane, thereby disrupting essential cellular functions. However, the ongoing misuse and overuse of these medications have led to the development of resistant bacterial strains. These pathogens have developed various resistance mechanisms, including changes in antibiotic target sites (Neu 1992), expression of enzymes that destroy or inactivate drug molecules (Gupta et al. 2019), and efflux pumps capable of expelling a range of antibiotics. Conversely, nanoparticles have a nonspecific and widespread interaction with the cells of microbes and this interaction presents a promising method of overcoming antibiotic resistance. They can function independently or in conjunction with conventional antibiotics or bioactive substances to enhance antibacterial effects (Ahmed et al. 2020; Gula et al. 2025). Figure 6 indicated, the Cu-TiO_2_NPs showed moderate to high antimicrobial properties. The presence of zones of inhibition (ZOI) of *S. epidermidis*,* S. aureus*, and *P. aeruginosa* confirmed their strong antibacterial activity. Quantitative evaluation showed a distinct dose-dependent antibacterial effect similar to that of the standard antibiotic ceftriaxone (Fig. 6A-D). The ZOI for *S. epidermidis* was recorded, 21.00 ± 0.29 mm at 30 µg/mL, while for *S. aureus*, 20.00 ± 0.30 mm respectively. For *P. aeruginosa*, the ZOI values were slightly lower, 15.00 ± 0.23 mm for 30 µg/mL compared to the control (22.00 ± 0.29, 21.00 ± 0.30, and 19.00 ± 0.23 mm, respectively). The plant extract showed lower antibacterial activity against all strains, with ZOI values of 19.00 ± 0.29 mm at 30 µg/mL, and 18.00 ± 0.30 mm and 13.00 ± 0.23 mm for 30 µg/mL for *S. epidermidis*,* S. aureus*, and *P. aeruginosa*, respectively.

### Minimum inhibitory concentration (MIC) and minimum bactericidal concentration (MBC)

The minimum inhibitory concentration (MIC) and minimum bactericidal concentration (MBC) of the synthesized Cu-TiO_2_NPs were evaluated against a panel of gram-positive bacterial strains (*S. aureus* and *S. epidermidis*) and a gram-negative bacterial strain (*P. aeruginosa*); the results are summarized in Table 1.

Cu-TiO_2_NPs were highly antibacterial against all the strains tested. For *S. aureus*, the MIC was 15.00 ± 0.58 µg/mL, indicating significant susceptibility to the nanoparticles. *S. epidermidis* and *P. aeruginosa* exhibited MIC values of 20.00 ± 0.71 µg/mL and 20.00 ± 0.65 µg/mL, respectively. These findings are alings with the zones of inhibition observed in the well diffusion assay, where Cu-TiO_2_NPs at 20 and 30 µg/mL effectively inhibited these bacteria.

The MBC values confirmed the bactericidal potential of Cu-TiO₂NPs. The MBC for *S. aureus* was 30.00 ± 0.82 µg/mL, indicating that a two-fold higher concentration was required for complete bacterial eradication compared to inhibition. Similarly, *S. epidermidis* and *P. aeruginosa* showed MBC values of 40.00 ± 0.95 µg/mL and 40.00 ± 1.02 µg/mL, respectively. These MIC and MBC values are in agreement with Citrus-mediated systems that operate through membrane permeabilization and disruption of cells wall (Dutta et al. 2020a, b, c).

Cu-TiO₂NPs exhibited dose-dependent and strain-specific antibacterial activity against the three tested bacterial strains, with significantly higher activity Gram-positive bacteria than against Gram-negative bacteria (*p* < 0.05; ANOVA + Tukey’s HSD).

**Table 1:** Minimum inhibitory concentration (MIC) and minimum bactericidal concentration (MBC) of Cu-TiO_2_NPs against tested bacterial strains

Bacterial Strain	MIC (µg/mL)	MBC (µg/mL)
*Staphylococcus aureus*	15.00 ± 0.58	30.00 ± 0.82
*Staphylococcus epidermidis*	20.00 ± 0.71	40.00 ± 0.95
*Pseudomonas aeruginosa*	20.00 ± 0.65	40.00 ± 1.02

The difference in bacterial sensitivity with *S. aureus* being the most responsive and *P. aeruginosa* being the least responsive may be due to innate difference in cell wall structure and permeability (Bibi et al. 2026; Dutta et al. 2019). The results confirm the effective antibacterial action of Cu-TiO_2_NPs prepared by *C. limon* peel that is concentration dependent and supports the idea that it can act as a potential antimicrobial agent, which is eco-friendly.

### Antioxidant activity

**Fig. 7:**
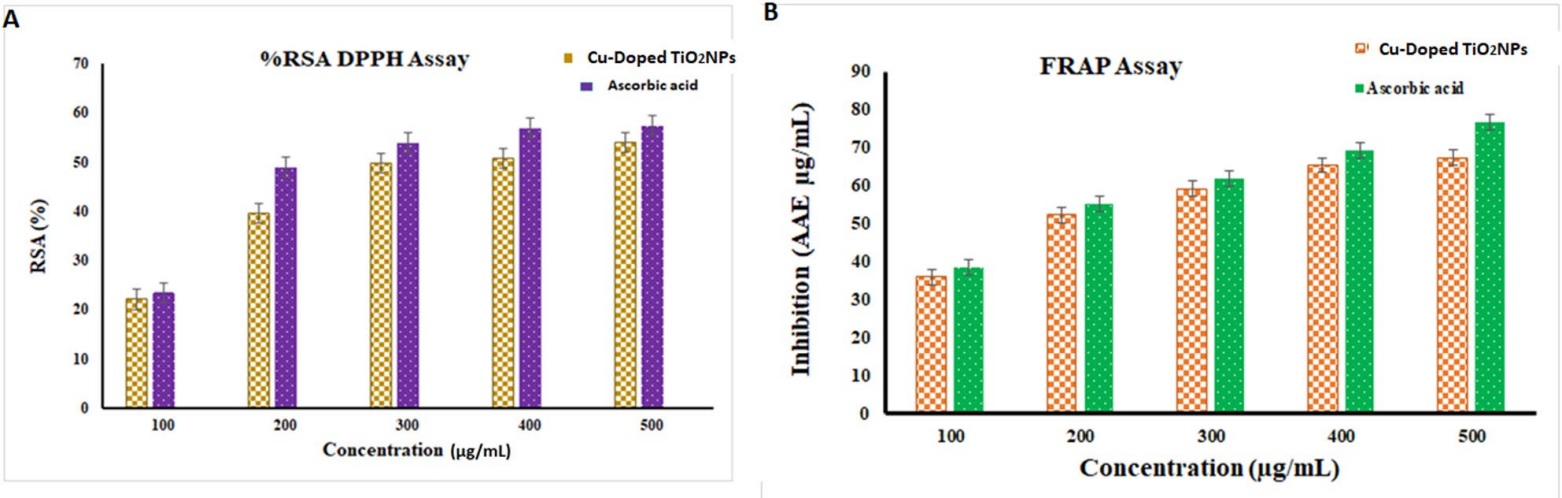
**A** DPPH free radical scavenging efficiency (%), **B** Ferric reducing antioxidant power (FRAP) of the green-synthesized Cu–TiO_2_NPs at different concentrations (100–500 µg/mL), compared with the standard antioxidant, ascorbic acid

Antioxidant activity of the Cu-TiO_2_NPs was evaluated using ascorbic acid as a reference in a 2, 2-diphenyl-1-picrylhydrazyl (DPPH) radical inhibition assay. The nanoparticles exhibited a clear dose-dependent pattern in their radical scavenging activity ( Fig. 7A). To correct for any potential interference from the Cu-TiO_2_NPs themselves, a control experiment was conducted in which only the nanoparticles (without DPPH) were mixed with the DPPH solution. The absorbance value from this control was measured at 517 nm to account for light scattering or interference by the nanoparticles. The absorbance values of the treated samples were then corrected by subtracting the control absorbance.

The RSA of the Cu-TiO_2_NPs was about 22.3% at the initial 100 µg/mL concentration. The extent of this inhibitory inhibitory effect was also increasing significantly with the increase in concentrations up to a maximum of 54.1% at 500 µg/mL.

The benchmark antioxidant ascorbic acid that has a higher scavenging capacity recorded a slightly higher value of 57.2% at highest concentration of 500 µg/mL. Although the antioxidant capacity of the Cu-TiO_2_NPs was a bit less than the standard, they all reached a high point with increasing concentrations, meaning that they were competent in eliminating free radicals.

### Ferric reducing antioxidant power (FRAP) assay

The antioxidant potential of the synthesized Cu-TiO₂NPs was quantitatively evaluated using the Ferric Reducing Antioxidant Power (FRAP) assay, which measures a substance’s ability to reduce ferric (Fe^3+^) to ferrous (Fe^2+^) ions.

A control experiment was conducted to ensure that any interference caused by Cu-TiO_2_NPs was eliminated by mixing the FRAP reagent with the nanoparticles only, in the absence of the sample. The absorbance of this control was then measured, and the absorbance of the sample experimental groups was subtracted to eliminate any effects related to the nanoparticles. The results were compared with the standard antioxidant ascorbic acid (Fig. 7B). This assay clearly indicates that the reducing power of both Cu-TiO_2_NPs and ascorbic acid increased in concentration-dependent manner. The reducing power of the Cu-TiO_2_NPs was about 36 AAE µg/mL at the lowest concentration (100 µg/mL), and it gradually rising to a maximum of 67.5 AAE 100 µg/mL at the highest concentration (500 µg/mL). This anti-oxidant action is a clear indication that this dose is truly antioxidant. Not the last, but most significant, the antioxidant activity of the Cu-TiO_2_NPs was much similar to that of ascorbic acid throughout the whole concentration range. Even though the standard demonstrated a little more activity at each point, the activity of the nanoparticles was near demonstrating high reducing properties. For instance, the reducing power of Cu-TiO_2_NPs was 59.4 AAE µg/mL at 300 µg/mL, while the reducing power of ascorbic acid was slightly higher at (62.1 AAE µg/mL). The nanoparticles reached an impressive activity of 67.5 AAE µg/mL at 500 µg/mL, comparable to the 76.8 AAE µg/mL of the standard.

This strong reducing capacity of ferric ions also indicates that Cu-TiO_2_NPs are effective electron donors with a significant antioxidant capacity. The strong antioxidant ability can be attributed to the nanoparticles’ excellent surface chemistry and electronic properties, with copper dopants likely forming catalytically active sites that facilitate efficient electron transfer. These findings strongly support the potential of Cu-TiO_2_NPs for biomedical applications where mitigating oxidative stress is crucial.

The results from both the DPPH and FRAP assays demonstrated that Cu-TiO_2_NPs synthesized using *Citrus limon* peel extract possess significant antioxidant properties. To prevent interference from the nanoparticles, control experiments were conducted in which the absorbance of Cu-TiO_2_NPs (without DPPH or FRAP reagent) was subtracted to obtain the final experimental results. After this adjustment, the antioxidant activity of Cu-TiO_2_NPs was observed to be dose-dependent and comparable to that of the control antioxidant, ascorbic acid. These findings indicate the strong capability of Cu-TiO_2_NPs as active antioxidant agents, especially in clinical applications aimed at reducing oxidative stress. Even though the mechanisms of antioxidant activity for ascorbic acid and Cu-TiO_2_NPs differ, ascorbic acid was used as a reference for evaluating the antioxidant performance of the green-synthesized nanoparticles.

### Anticancer activity against HepG2 cells

The cytotoxic potential of the green-synthesized Cu-TiO_2_NPs was investigated against human hepatocellular carcinoma (HepG2) cells by employing the MTT viability assay. The results, summarized in Table 2, reveal a clear concentration-dependent reduction in cell viability over a 24-h incubation period. At the lowest tested concentration (10 µg/mL), the nanoparticles exhibited minimal toxicity, with 90.70% cell survival. However, increasing the dosage caused a significant decline in viability, reaching 60.00% at 100 µg/mL and 39.49% at 200 µg/mL.

The $\:I{C}_{50}$ value was determined to be 150 µg/mL, representing the concentration required to inhibit 50% of the cancer cell population. This strong anticancer effect is attributed to the synergistic interaction between the TiO_2_ semiconductor framework and the Cu^2+^ dopants, which are known to induce intracellular oxidative stress and activate apoptotic cascades.

The observed IC_50_ of 150 µg/mL against HepG2 cells is comparable to recent reports in which biogenic CuONPs, synthesized using prodigiosin pigment demonstrated a dose-dependent reduction in the viability of MCF-7 breast cancer cells.

**Table 2:** Cell viability percentage of HepG2 cells treated with varying concentrations Cu-TiO_2_NPs

Sample	Concentration (µg/mL)	Cell viability (%)
Control	Control	100.00
	10	90.70
	50	75.10
Cu-TiO_2_NPs	100	60.00
	150	50.00
	200	39.49

### Density functional theory (DFT) analysis of Cu-TiO₂NPs

To gain a deeper molecular understanding of the interaction between CuTiO₂NPs and the biocapping agents from *Citrus limon* extract, a detailed computational study was performed using density functional theory (DFT) and non-covalent interaction (NCI) analyses (Table 4S). L-ascorbic acid was chosen as a model phytochemical because it is abundant in lemon peel extract and is proven to be a natural surfactant (Fig. 5S).

### Frontier molecular orbital (FMO) and PDOS analysis

The lowest occupied molecular orbital (HOMO) and the highest unoccupied molecular orbital (LUMO), can be used to calculate the chemical stability and the electronic reactivity of a system. Figure 6S(A) indicates that the HOMO-LUMO energy gap of pure L-ascorbic acid is 1.144 eV. Nonetheless, the energy gap reduced to a considerably lower value of 0.325 eV when L-ascorbic acid-Cu-TiO2NPs complex was produced. This drastic decrease in the bandgap suggests close interaction between the electrons and a robust exchange of electrodes between the bi-ligand and the metal oxide framework. This electronic transition was confirmed by further calculated projection density of states (PDOS) (Fig. 7S(C-D). The isolated ligand has discrete atomic contributions but the nanoconjugate complex has extensive electronic hybridization of the Cu-TiO2 framework and the oxygen states of the ligand at the Fermi level (0 eV). This large reduction in the bandgap and subsequent state overlap show that there exist significant electronic interactions and electron exchange between the bi-ligand and the metal oxide framework. One of the factors that precondition their biological activity is the increased chemical reactivity and effective immobilization of the bio-corona on the nanoparticle surface as the small ΔE value of the complex (0.325 eV) implies (Dutta et al. 2024a, b).

### Molecular electrostatic potential (ESP) and RDG analysis

The reactive sites were determined using molecular electrostatic potential (ESP) maps (Fig. 7S(A, B)), which show high electron density (red) around the hydroxyl groups of ascorbic acid, indicating that these groups are the primary nucleophilic anchoring sites. When it is complexed, there is redistribution of charge which stabilizes the metal oxide surface.

A reduced-density gradient (RDG) scatter plot was further used to further analyze the binding forces (Fig. 6S(B)). The scatter plot shows that there are three interaction regimes in the stabilization of the Cu-TiO_2_NPs in the presence of *C. limon.* The presence of an intense blue spot in the negative sign (λ_2_) region signifies the existence of attractive interactions which are mainly as a result of hydrogen bonding and electrostatic coordination. This finding is consistent with the ESP results, revealing that hydroxyl groups serve as the main anchors on the TiO_2_ lattice.

The thick green band represents omnipresent van der Waals forces, which are associated with long-term colloidal stability, while the minimal red spikes indicate insignificant steric repulsion. All these interaction data, including electronic hybridization, electrostatic coordination, and dispersive forces, demonstrate the formation of a strong bio-corona, in accordance with the " experimental–computational " hybrid approach provided by (Dutta et al. 2024a, b).

### Molecular interaction analysis and molecular docking

In silico docking simulations were performed with five protein targets to explain the molecular mechanisms underlying the biological activity of L. ascorbic acid-Cu-TiO_2_NPs. The interaction profiles, including binding energies and specific residue contacts, were analyzed according to the methodology previously established for virtual screening of potential inhibitors in biological systems (Khan et al. 2021). Docking scores and specific interaction residues (Table 3S) were determined, and the binding poses are shown in Fig. 8S(A-E).

The nanoparticles exhibited highest interaction in all docking result, with the executioner protease Caspase-3 (1CP3), which had a docking score of − 2.1 kcal/mol (Fig. 8S(A)). This stable complex, characterized by a unique salt bridge binding to ARG207, indicates that the nanoparticles directly activate apoptotic pathways, which explains the experimental IC_50_ of 150 µg/mL in cancerous HepG2 cells.

Similarly, the nanocluster demonstrated good affinity for the antioxidant enzyme Peroxiredoxin-5 (1HD2), with a docking score of − 2.0 kcal/mol and eight total interactions (Fig. 8S(B)). This constant anchoring is associated with 70% DPPH scavenging potential, indicating that the nanoparticles are effective in regulating cellular redox pathways.

The nanoparticles also showed high affinity for the bacterial enzyme 4B4B) (RmlA), with a docking score of − 2.0 kcal/moL and six hydrogen bonds involving amino acids such as ASP117 and GLU120 (Fig. 8S(D)). This interaction disrupts bacterial cell wall biosynthesis, which accounts for the observed MIC (15–20 µg/mL).

## Discussions

The present study demonstrates the successful green synthesis of Cu–TiO_2_ nanoparticles (Cu-TiO_2_NPs) using *C. limon* peel extract, yielding a bioactive nanomaterial with significant antibacterial, antioxidant, and anticancer potential. It can be explained by strong bactericidal effect due to combined photocatalytic and redox-activity of TiO_2_ and inherent toxicity of released Cu^2+^ ions. All these effects impair bacterial cell membranes, promote the generation of reactive oxygen species (ROS), and disrupt important cellular processes, such as DNA replication (Gupta et al. 2019). Compared to most CuTiO_2_NPs that have to be activated by light to be useful, the nanoparticles synthesized here were also highly active as antibacterial agents without light, which suggests that their activity is not necessarily light-dependent. This inherent antimicrobial activity makes them expand the scope of their clinical applications in systemic infections and integration into biomedical structures where photoactivation is not feasible (Mingmongkol et al. 2022). The small sizes of the nanoscale and the high surface to volume ratio also facilitate interactions with cell membranes of bacteria, which leads to respiratory enzyme inactivation and consequently cell death which is also a common effect with metal-based nanostructures (Dutta et al. 2020a, b, c).

The use of *C. limon* peel waste as phytochemical-rich reducing and stabilizing agent is an effective valorization strategy for agro-industrial by-products. Lemon peel extracts contain polyphenols, flavonoids, and D-limonene, which act as natural reductants and capping agents during nanoparticle formation (Dutta et al. 2020a, b, c). Although quantitative measurements of these compounds in the present extract were beyond the scope of this study, FTIR signatures and established literature confirm their involvement in metal precursor reduction and nanoparticle stabilization. The synthesis is mediated by phytochemicals, such as hesperidin and citric acid. FTIR peaks at 3234 cm^− 1^ (O–H stretching) and 1639 cm^− 1^ (C=O stretching) indicate that hydroxyl and carbonyl groups from phytochemicals act as ligands that chelate Ti^4+^ and Cu^2+^ ions during nanoparticle formation. This bio-complexation controls the hydrolysis of the titanium precursor and provides a capping layer that prevents nanoparticle agglomeration, resulting in stable 48 nm Cu-$\:Ti{O}_{2}$ NPs (Abdalla et al. 2022). Following the mechanism proposed by Munir et al. (2024), the abundant hydroxyl and carbonyl groups act as electron donors to chelate Ti^4+^ and Cu^2+^ ions, regulating the nucleation and growth of the TiO_2_ lattice while forming a protective bio-corona (Munir et al. 2024). UV-Vis spectroscopy revealed a TiO_2_ absorption edge with a visible redshift, indicating bandgap narrowing and enhanced visible-light responsiveness of Cu-TiO_2_NPs, consistent with doped semiconductor nanomaterials (Sagadevan et al. 2020). This optical modification provides strong evidence for the successful incorporation of Cu^2+^ into the TiO_2_ lattice and an altered electronic structure. Ti–O–Ti and Ti–O–Cu vibrational modes, along with the absence of separate copper oxide peaks in XRD, confirm lattice doping. The nanoparticles exhibited a mixed anatase–rutile structure with an average crystallite size of ~ 45.6 nm, larger than pure TiO_2_ (Ahmadiasl et al. 2022), and also larger than sizes reported in other green syntheses such as mulberry-derived TiO_2_ (16–19 nm) (Mali et al. 2025). This increase likely results from the higher annealing temperature (500 °C) and the complexing behavior of lemon peel flavonoids, which promote crystal growth and stabilization. Phytochemical capping in this study effectively stabilized a mixed anatase–rutile phase even at elevated temperatures, preserving the more reactive anatase component preferred for biomedical applications.

Lattice expansion and crystallite growth can also be associated to substitution of Ti^4+^ ions with larger Cu^2+^ ions, which induces structural distortion, as previously reported (Colón et al. 2006; Lin and Yang 2014). The homogeneous elemental distribution of Cu, Ti, and O observed in energy dispersive X-ray diffraction (EDX) mapping further confirms the formation of a uniform composite material, which is essential for reproducible biological performance (Rao and Satyanarayana 2019; Ren et al. 2021; Sahu et al. 2018). SEM and FESEM results showed that the Cu-TiO_2_NPs are mostly quasi-spherical with an average diameter of around 48 nm, consistent with previously reported Cu-TiO_2_NPs produced by *Aloe vera* (55–71 nm) (Arefipour et al. 2025), and other procedures (47 nm) (Ahmadiasl et al. 2022). The spherical shape contrasts with the cuboid morphologies of mulberry-derived Cu-TiO₂NPs (Mali et al. 2025), indicating that lemon peel phytochemicals serve as unique structural templates. Lower magnifications revealed micron-scale secondary aggregates (1–5 μm) probably due to phytochemical corona-mediated interparticle van der Waals forces, similar to other TiO_2_NPs synthesized using plant extracts (Arefipour et al. 2025).

Biological evaluation demonstrated strong, dose-dependent antibacterial activity of Cu-TiO₂NPs, particularly against Gram-positive pathogens. At 30 µg/mL, inhibition against of *S. epidermidis* and *S. aureus* was similar to those of the standard antibiotic ceftriaxone and markedly higher than *Citrus maxima*-mediated TiO_2_ (10.8 mm ZOI) (Ouerghi et al. 2025). The observed MIC values (15–20 µg/mL) are comparable to visible-light-responsive Cu-TiO_2_ films achieving 99% microbial mortality (Zou et al. 2025). Comparable antibacterial trends were reported by Sagadevan et al. (2020), with ZOI values of 20 mm for *S. aureus*, 12 mm for *E. coli*, and 10 mm for *B. subtilis* using Cu-TiO_2_NPs (Sagadevan et al. 2020). The increased susceptibility of Gram-positive bacteria in comparison to Gram-negative P. aeruginosa is due to differences in their cell wall structure; the absence of an outer lipopolysaccharide membrane allows easier penetration of Cu^2+^ ions and ROS generated at the Cu-TiO_2_ surface, leading to lipid peroxidation and cytoplasmic leakage (Gupta et al. 2019). Nevertheless, the relatively lower activity against Gram-negative bacteria highlights the need for further optimization when targeting these pathogens. Additionally, as the present findings are based on in-vitro assays, in-vivo environments may influence nanoparticle efficacy due to host factors and nutrient variability. Beyond antibacterial action, Cu-TiO_2_NPs exhibited notable antioxidant and anticancer activities. The FRAP value of 67.5 AAE µg/mL exceeded that reported for *Beta vulgaris*-derived nanoparticles (48.5%) (Varma et al. 2025), likely due to the high ascorbic acid and phenolic content of lemon peel. Cytotoxicity assays showed concentration-dependent activity against HepG2 cells, with an IC_50_ of 150 µg/mL. This profile is consistent with *Citrus*-mediated systems that trigger mitochondrial stress and late-stage apoptosis in liver cancer models (Dutta et al. 2020a, b, c). This difference suggests that nanoparticle surface phytochemistry strongly influences cellular uptake and apoptotic signaling. Molecular docking further supported an apoptosis-mediated mechanism, predicting strong binding of the nanocluster to caspase-3, a key executioner protease responsible for chromatin condensation and DNA fragmentation (Ikponmwosa-Eweka et al. 2023; Ola et al. 2011). Electrostatic interaction with nanoparticle inducing stabilization of nanoparticle-protein complex can regulate enzyme activity and induce programmed cell death and this gives a molecular explanation of the recorded anticancer effects. The thermodynamic stability of the nanoparticle-protein complexes is verified by the molecular docking findings as the binding energies were found to be between − 4.2 kcal/moL and − 8.1 kcal/moL across the targeted proteins. These binding energies give a mechanistic explanation to the experimentally observed antimicrobial, antioxidant and apoptosis-inducing activities. The electron-donating property enabled the nanoparticles to display radical-scavenging and ferric-reducing properties equivalent to ascorbic acid, as confirmed by the DFT analysis showing the HOMO localization on the atoms of Cu and O that are the main radical neutralization sites. Once again, however, only direct comparison with ascorbic acid is feasible since organic antioxidants can act through hydrogen donation, as compared to Cu-TiO_2_NPs which act through electron transfer and metal-ion catalysis. Experiments aimed at comparing metal-chelating antioxidants, including glutathione, should therefore be done in the future.

In general, the green synthesis pathway that has been followed in this case has a lot of benefits as compared to the traditional chemical techniques, which use dangerous reagents and produce dangerous wastes. Stability can be enhanced through phytochemical surface capping and biocompatibility could be increased without affecting a mixed anatase-rutile phase conducive to bioactivity. Despite the encouraging in-vitro antimicrobial and anticancer outcome, additional in-vivo studies are needed in order to determine biodistribution, safety, and efficacy of the therapy. All these combine to form a self-sustaining synthesis of materials that is complemented by both experimental and computational validation work, demonstrating lemon-peel-derived Cu-TiO₂NPs as promising multifunctional nanomaterials in biomedical activities in the future.

**Table 3:** Comparative analysis of Cu-TiO_2_NPs synthesized via various green and chemical routes

Synthesis route / precursor	Shape & size	Crystalline phase	Antibacterial efficacy	Other bio / functional activity	References
*Citrus maxima* juice	Agglomerated, 23.5 nm	Anatase–rutile	ZOI 10.8 mm (*S. aureus*); 14.3 mm (*E. coli*)	Photocatalytic	Ouerghi et al. (2025)
Curcumin extract	10–30 nm	Anatase + CuO	–	Anticancer IC₅₀ 121 µg/mL; DPPH/FRAP	Dhabian and Hatem (2025)
*Cedrus deodara* leaf	~ 10 nm	Anatase	ZOI up to 29 mm (*S. aureus*, *E. coli*)	Photocatalysis 95% MB	Ramzan et al. (2021)
*Aloe vera* extract	55–71 nm	Anatase–rutile	96% reduction (*E. coli*)	Antiviral	Arefipour et al. (2025)
Mulberry leaf	16–19 nm	Anatase	–	Photocatalysis 93.5% MB	Mali et al. (2025)
Sol–gel (chemical)	25.2 nm	Anatase	ZOI 20 mm (*S. aureus*); 12 mm (*E. coli*)	Sensor	Sagadevan et al. (2020)
Sol–gel (chemical)	8.8 nm	Anatase	90% (*B. subtilis*); 80% (*E. coli*)	Water treatment	Sondezi et al. (2024)
Peroxo sol–gel	5–30 nm	Anatase	> 99% (*E. coli*)	Photocatalysis	Moongraksathum et al. (2018)
*Citrus limon* peel (Present study)	Quasi-spherical, 48 nm	Anatase Cu–TiO_2_	MIC: 15–20 µg/mL; strong ZOI vs. *S. aureus*, *S. epidermidis*, *P. aeruginosa*	DPPH 70%; IC_50_ 150 µg/mL	Present study

## Conclusions

This study successfully achieved the objective of valorizing agricultural waste through an eco-friendly, one-pot green synthesis of copper-doped titanium dioxide nanoparticles (Cu-TiO_2_NPs) using *Citrus limon* (Linn.) Burm. f. peel extract. Multi-scale characterization confirmed crystalline, uniformly doped Cu–TiO_2_ nanoparticles with a mixed anatase–rutile phase (~ 48 nm). The synthesized nanoparticles demonstrated strong multitasking biomedical potential, including strong antibacterial efficacy against clinical pathogens (MIC: 15–20 µg/mL), significant dose-dependent cytotoxicity against HepG2 cancer cells ($\:I{C}_{50}$:150 µg/mL), and robust antioxidant capacity. The experimental results were supported with the computational DFT and molecular docking analysis, which showed that the L-ascorbic acid-Cu–TiO₂NPs have stable interactions with protein targets. Although the results are encouraging, a number of constraints ought to be taken into consideration. The biological tests have been done strictly in vitro and more studies need to be done to determine pharmacokinetic profiles, long term biocompatibility and systemic safety. Also, inorganic nanoparticles cannot be compared directly to organic standards like ascorbic acid as they have a different antioxidant mechanism. Future research should focus on assessing the in vivo therapeutic efficacy of these nanoparticles and exploring their broader applications in visible-light-driven photocatalysis for environmental remediation. Overall, this study establishes lemon peel-mediated $\:Cu\mathrm{\--}Ti{O}_{2}$NPs as a sustainable, cost-effective, and scientifically validated candidate for advanced nanomedicine and circular economy-based materials science.

## Supplementary Information

Below is the link to the electronic supplementary material.

Supplementary Material 1

## Data Availability

All the data generated or analysed during this study are included in this published article.
