# Supplementary material for: Eco-friendly Cu–TiO2 nanoparticles from Citrus limon peel: integrated biological and computational evaluation
Source: AMB Express. 2026 May 2;16:70. doi: 10.1186/s13568-026-02060-2 (PMC13315372; doi:10.1186/s13568-026-02060-2)
Supplement: Supplementary file 1 — Supplementary Material 1 [file 13568_2026_2060_MOESM1_ESM.docx]

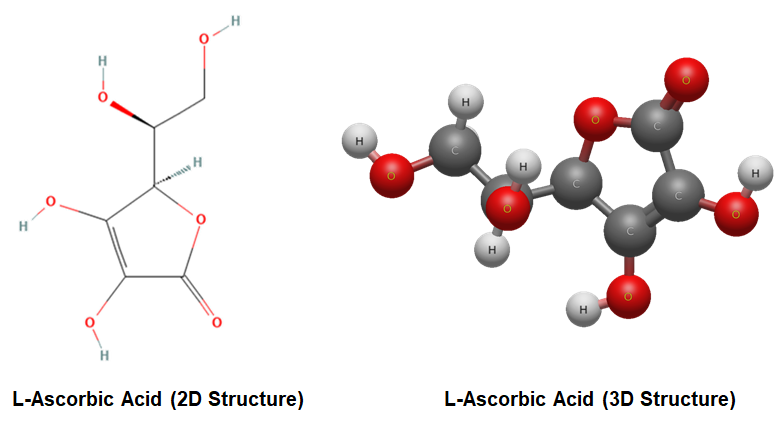


**Figure. 1S**  Structure of L-Ascorbic Acid


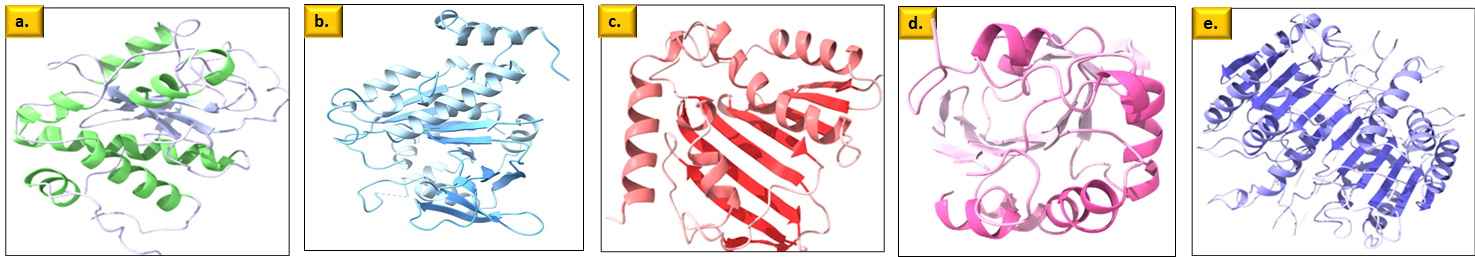
 **Figure 2S. (a**) atlE (*PDB ID: 3LAT)* - *S. epidermidis* (**b**) RmlA *(PDB ID: 4B4B)-P. aeruginosa* (**c**) *GYRB (PDB ID: 3TTZ)-S. aureus* (**d**) Anti-oxidant PRDX5 (PDB ID: 1HD2*)* *Homo sapiens,* (e) Anticancer protein Caspase-3 (PDB ID: 1CP3)


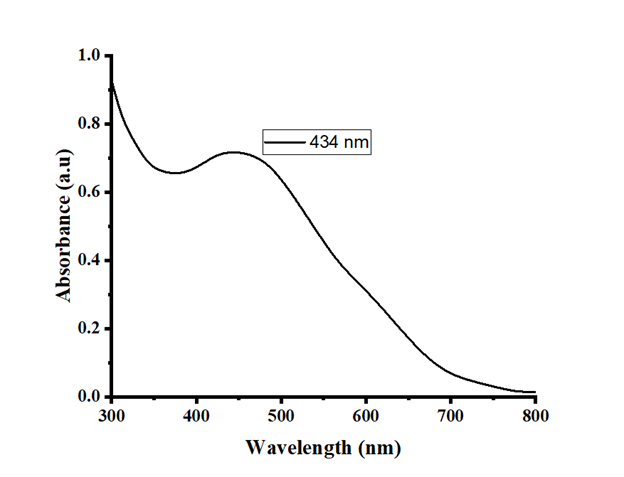
 **Figure 3S.** UV-visible spectra of biosynthesized Cu-TiO_2_NPs


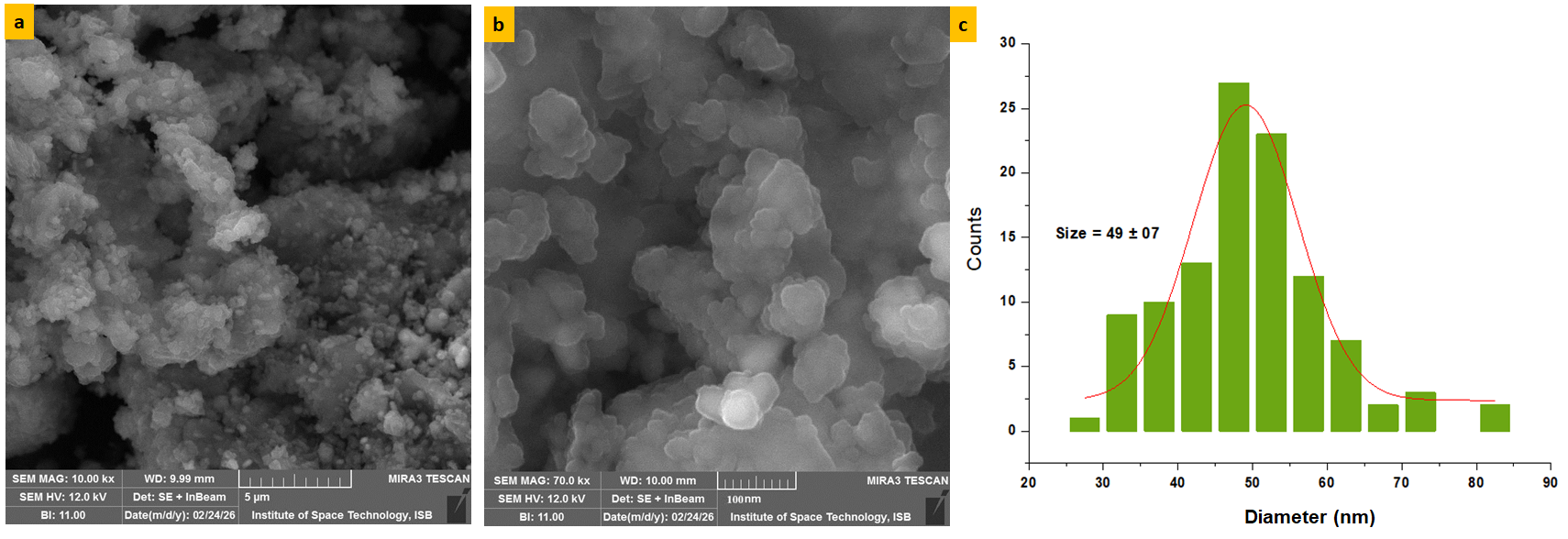
**Figure 4S.** (a-b) Field Emission Scanning Electron Microscopy (FESEM) Analysis at different magnifications, (c) Histogram of Cu-TiO_2_NPs


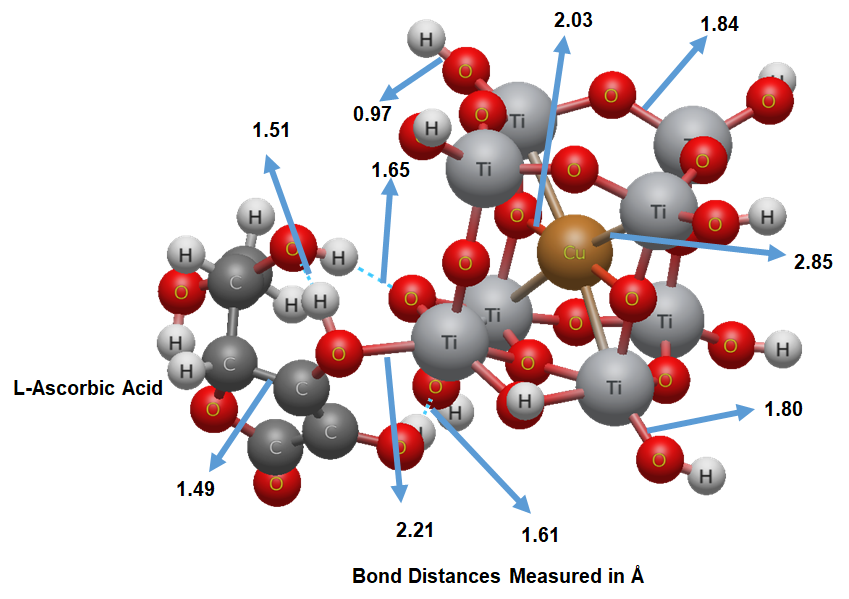


**Figure. 5S** Optimized structures of Cu-TiONPs - L-Ascorbic Acid Complex.

**Table 1S.** Custom Lennard-Jones parameters for Cu(II) and Ti(IV) coordination in AutoDock 4.2.

| Atom Type | Rii (Å) | | εii (kcal/mol) | | Description | |
| --- | --- | --- | --- | --- | --- | --- |
| Cu | 1.50 | | 0.150 | | Copper(II) ion | |
| Ti | 1.60 | | 0.120 | | Titanium(IV) ion | |
| NM | 2.50 | | 0.160 | | Cu-coordinating nitrogen | |
| OM | 2.40 | | 0.200 | | Cu-coordinating oxygen | |
| SM | 2.80 | | 0.200 | | Cu-coordinating sulfur | |
| NT | 2.60 | | 0.160 | | Ti-coordinating nitrogen | |
| OT | 2.30 | | 0.200 | | Ti-coordinating oxygen | |
| Validated Coordination Distances | | | | | | |
| Metal–Ligand | | **Calculated (Å)** | | **Target (Å)** | | **Reference** |
| Cu–NM | | 2.00 | | 2.00 | | ([Rulı́šek & Vondrášek, 1998](#_ENREF_15)) |
| Cu–OM | | 1.95 | | 1.95 | | ([Harding, 2001](#_ENREF_7)) |
| Cu–SM | | 2.15 | | 2.15 | | ([Rulı́šek & Vondrášek, 1998](#_ENREF_15)) |
| Ti–NT | | 2.10 | | 2.10 | | ([Rappé et al., 1992](#_ENREF_12)) |
| Ti–OT | | 1.95 | | 1.95 | | ([Rappé et al., 1992](#_ENREF_12)) |

Note: Distances calculated using Lorentz-Berthelot combining rule: Rij = (Rii + Rjj) / 2, All other parameters retained from AutoDock.


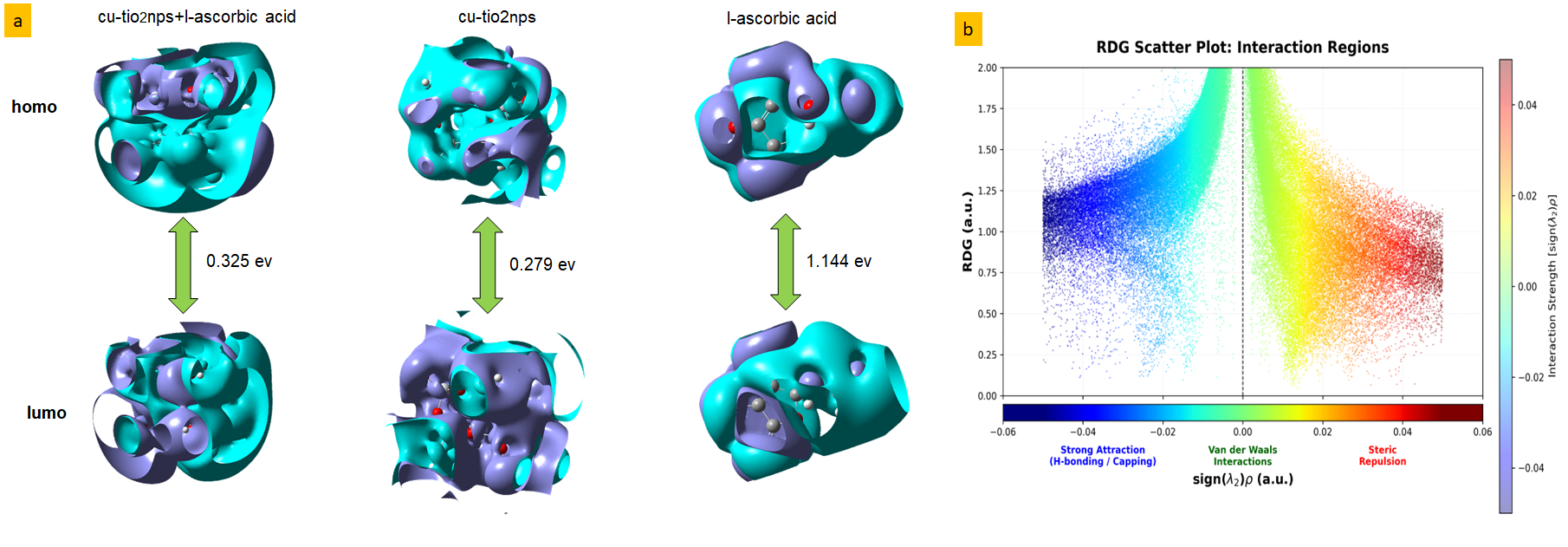


**Figure 6S**. (A) HOMO-LUMO calculation (B) Reduced density gradient (RDG) of L-ascorbic acid-Cu-TiO_2_NPs


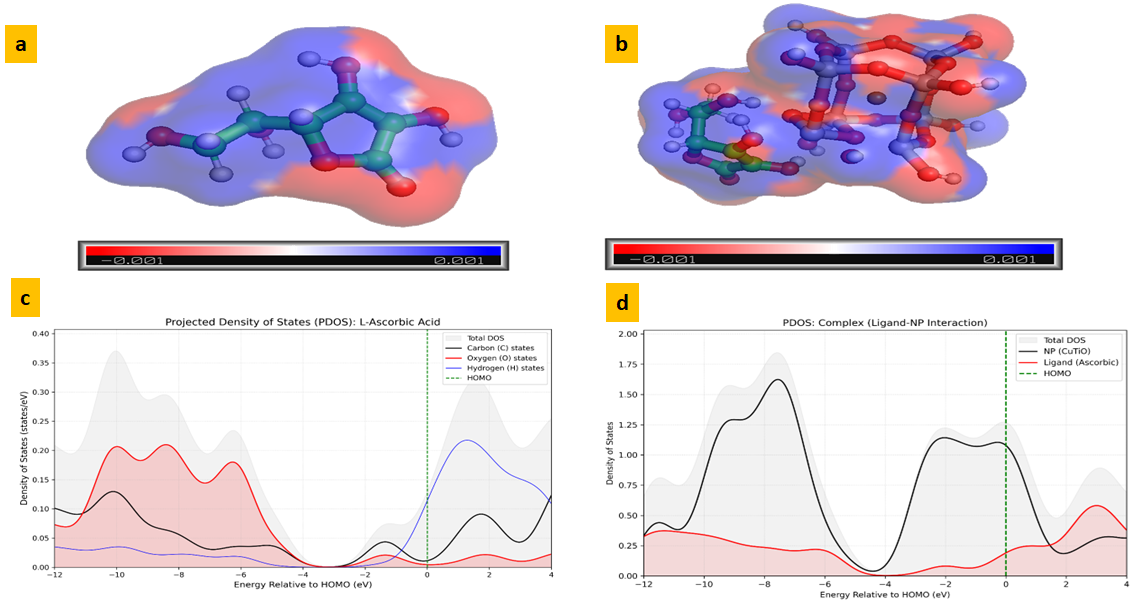


**Figure 7S.**ESP and PDOS analysis of isolated L-ascorbic acid and (B, D) L.ascorbic acid-Cu-TiO_2_NPs illustrating surface coordination and electronic coupling

**Table 2S.** Grid center coordinates (Å) used for docking, positioned to cover the active sites of Autolysin E (3LAT), RmlA (4B4B), DNA Gyrase B (3TTZ), Peroxiredoxin-5 (1HD2) and Caspase-3 (1CP3).

| Protein (PDB ID) | Target Description | Grid Center X (Å) | Grid Center Y (Å) | Grid Center Z (Å) |
| --- | --- | --- | --- | --- |
| 3LAT | Autolysin E | 3.8 | 65.7 | 90.5 |
| 4B4B | RmlA | 34.4 | -21.1 | 55.8 |
| 3TTZ | DNA Gyrase B | 0.0 | 2.7 | 24.4 |
| 1HD2 | Peroxiredoxin-5 | 21.2 | 41.2 | 13.7 |
| 1CP3 | Caspase-3 | 27.7 | 11.2 | 37.6 |

**Table 3S.** Docking scores of L-Ascorbic acid-Cu-TiO_2_NPs with targeted proteins

| Protein (PDB ID) | Target | Docking scores | Hydrogen Bond Residues (No.) | Salt Bridge Residues (No.) | H-Bond Distance Range (Å) | Total Interactions |
| --- | --- | --- | --- | --- | --- | --- |
| 3LAT | Autolysin E | ~ -1 | ASN64 (1), GLU72 (1), ASN82 (1), HIS177 (1) | — | 3.01–3.70 | 4 |
| 4B4B | RmlA | ~ - 2 | GLY115 (1), ASP117 (1), PHE118 (1), GLU120 (1), ALA251 (1), VAL292 (1) | — | 2.60–3.68 | 6 |
| 3TTZ | DNA Gyrase B | ~ -1 | GLU58 (2), ARG84 (2) | — | 2.21–3.37 | 4 |
| 1HD2 | Peroxiredoxin-5 | -2.0 | GLU16 (2), GLY85 (1), ARG86 (3), GLU91 (1), LEU96 (1) | — | 2.57–3.93 | 8 |
| 1CP3 | Caspase-3 | -2.1 | THR62 (2), ARG64 (1), HIS121 (1), ARG207 (2) | ARG207 (1) | 2.51–3.36 | 7 |

**Table 4S.** XYZ Cartesian coordinates (Å) for the DFT-optimized L-ascorbic acid-Cu-TiO₂NPs nanoconjugate**.**

| Atom | X | Y | Z |
| --- | --- | --- | --- |
| O | 4.42729334 | 1.84850270 | 4.45245650 |
| O | -2.10312560 | 2.16288692 | 3.07508927 |
| O | -1.07505680 | 1.82298168 | 5.68826047 |
| O | 0.11764645 | 1.92757669 | 2.21049334 |
| H | -0.35036546 | -0.15918896 | 0.58427454 |
| O | -1.72270681 | 4.23051626 | 1.13372137 |
| O | -2.77514826 | 6.80910091 | 0.00541585 |
| O | 4.37615942 | 6.89062587 | 3.90066989 |
| O | -0.38518243 | 4.32470021 | 4.05368098 |
| O | -1.20479196 | 6.70290506 | 2.53811188 |
| O | -0.28652031 | 2.34671796 | -0.23979394 |
| O | 1.17969218 | 0.14535889 | 0.15798275 |
| O | 1.43328835 | 2.12767543 | 4.46778005 |
| O | -1.14683297 | 6.86704980 | 5.53252884 |
| O | 2.83974215 | 1.73991038 | 1.91373988 |
| O | 0.17674722 | 6.25824206 | -0.03222375 |
| O | 1.66555123 | 3.88209847 | 0.64518530 |
| O | 2.72326630 | 5.65992053 | -1.51058422 |
| O | 1.38731270 | 6.69281290 | 4.03098198 |
| O | 2.98943106 | 4.30030026 | 3.37492284 |
| O | 2.76784409 | 6.44037767 | 1.41999074 |
| Ti | -1.45872645 | 2.43848898 | 1.14857394 |
| Ti | -0.33649296 | 2.44716155 | 4.16062940 |
| Ti | -1.39536590 | 6.03382638 | 0.87268936 |
| Ti | -0.33205820 | 6.19950771 | 4.06928906 |
| Ti | 1.35438522 | 1.92301217 | 0.81373624 |
| Ti | 2.98689200 | 2.47174368 | 3.56270700 |
| Ti | 1.85746275 | 5.58397380 | 0.06943257 |
| Ti | 2.91794728 | 6.11122020 | 3.18545910 |
| Cu | 0.64048102 | 4.18004643 | 2.31272848 |
| O | -2.22426879 | -0.51682440 | -2.76187670 |
| O | -1.83971126 | 3.06903236 | -2.23294979 |
| O | -3.03558652 | 2.05241528 | -0.34783533 |
| O | -0.53020873 | 0.60009094 | -4.62767090 |
| O | -1.36905334 | -0.04818817 | 0.72111506 |
| O | -0.93841881 | -2.02725760 | -1.61882600 |
| C | -2.79525221 | 0.78101901 | -2.49751239 |
| C | -1.91945697 | 1.95297003 | -3.13222348 |
| C | -2.71149204 | 0.89838647 | -1.01151547 |
| C | -0.51185982 | 1.46852895 | -3.52536017 |
| C | -1.91964916 | -0.07822118 | -0.51429549 |
| C | -1.60636931 | -1.02267476 | -1.60350459 |
| H | -3.82317112 | 0.79193148 | -2.91579998 |
| H | -2.42721530 | 2.30638840 | -4.05192901 |
| H | 0.06105764 | 2.37521991 | -3.81475219 |
| H | -0.00020424 | 1.04166563 | -2.62684529 |
| H | -1.06039092 | 2.89683152 | -1.59301625 |
| H | -2.78315083 | 2.76842135 | -1.08622550 |
| H | -0.95924106 | -0.22078274 | -4.31016576 |
| H | -3.44834498 | 7.39973642 | 0.38801611 |
| H | 2.45798091 | 6.06057138 | -2.35634187 |
| H | 4.73074281 | 2.06393017 | 5.35149927 |
| H | -0.52478237 | 1.63619229 | 6.47163941 |
| H | -0.71500866 | 7.23496920 | 6.32429383 |
| H | 5.30202405 | 6.86072281 | 3.60210177 |
| H | 1.83030548 | -0.53190631 | 0.42470891 |
| H | -2.85700244 | 2.67363444 | 3.42751458 |


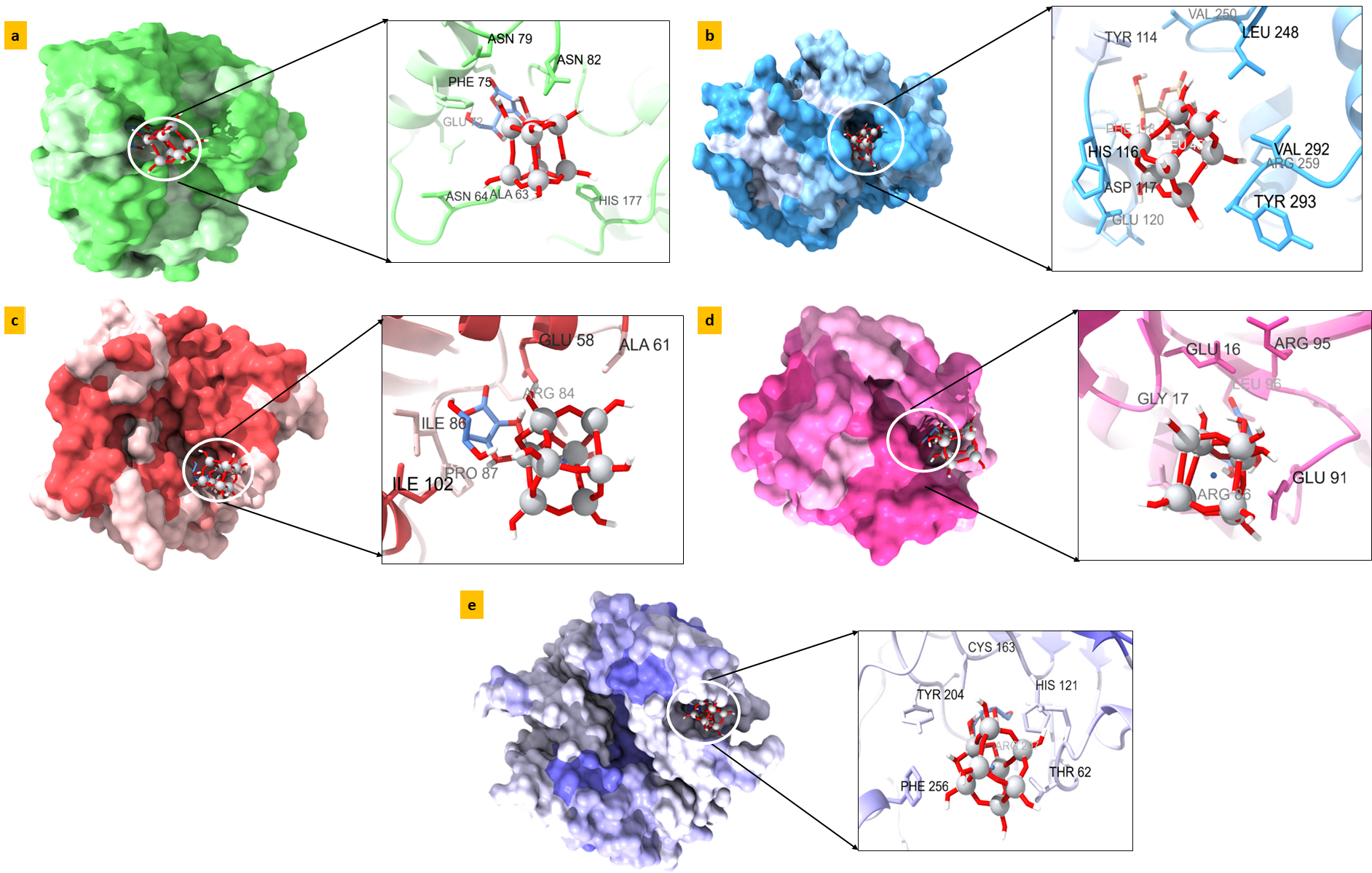


**Figure 8S. (a**) atlE (PDB ID: 3LAT) - *S. epidermidis* (**b**) RmlA (PDB ID: 4B4B)-*P. aeruginosa* (**c**) *GYRB* (PDB ID: 3TTZ)-*S. aureus* (**d**) Anti-oxidant PRDX5 (PDB ID: 1HD2*)* *Homo sapiens,* (**e**) Anticancer protein Caspase-3 (PDB ID: 1CP3)
